# Supplementary material for: PBAF/cBAF reorganization on H3.3 chromatin regulates BMAL1 activity in the absence of circadian negative feedback
Source: Nat Commun. 2025 Oct 9;16:9000. doi: 10.1038/s41467-025-64045-2 (PMC12511354; doi:10.1038/s41467-025-64045-2)
Supplement: Supplementary file 1 — Supplementary Information [file 41467_2025_64045_MOESM1_ESM.pdf]

PBAF/cBAF reorganization on H3.3 chromatin regulates BMAL1 activity in the absence of circadian negative feedback

List of Supplementary Materials:

Figures

Supplementary Figure 1. Native purification of H3.3A liver protein complexes.

Supplementary Figure 2. H3.3A signal at genes near CLOCK-BMAL1 binding sites normalized to the input.

Supplementary Figure 3. H3.3A occupancy dynamics at intergenic regions and CLOCK-BMAL1 target genes.

Supplementary Figure 4. H3.3A enrichment on wild-type vs. PerKO chromatin.

Supplementary Figure 5. Histone marks and variants in PBAF complexes, and expression pattern of subunits.

Supplementary Figure 6. Expression of H3.3A and chaperones.

Supplementary Figure 7. Impact of H3.3 loss on clock gene expression.

Supplementary Figure 8. BMAL1 presence on chromatin and higher turnover in absence of PER.

Supplementary Figure 9. Impact of specific PBAF and cBAF components on clock gene expression.

Supplementary Figure 10. Reanalysis of published datasets for PBAF and cBAF specific components siRNA screens.

a

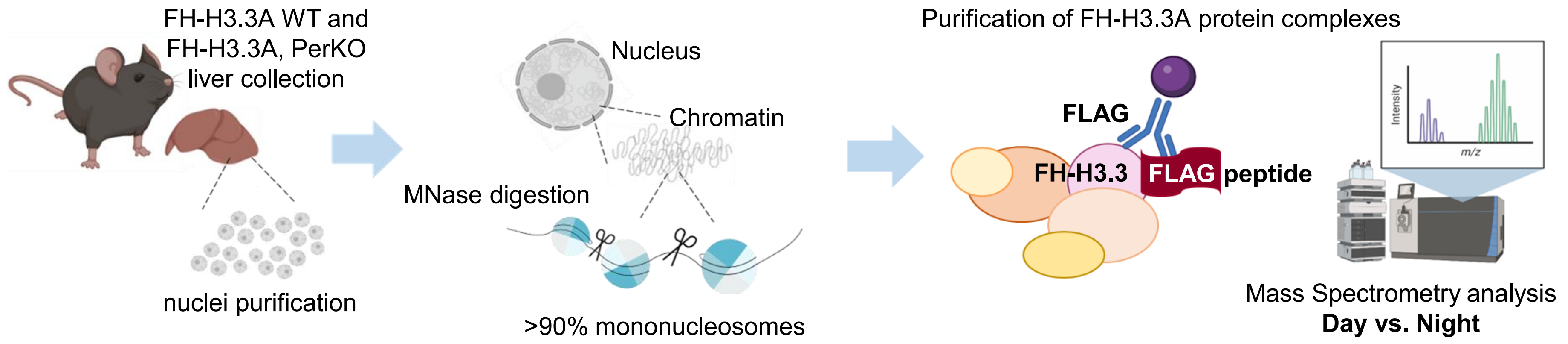

b

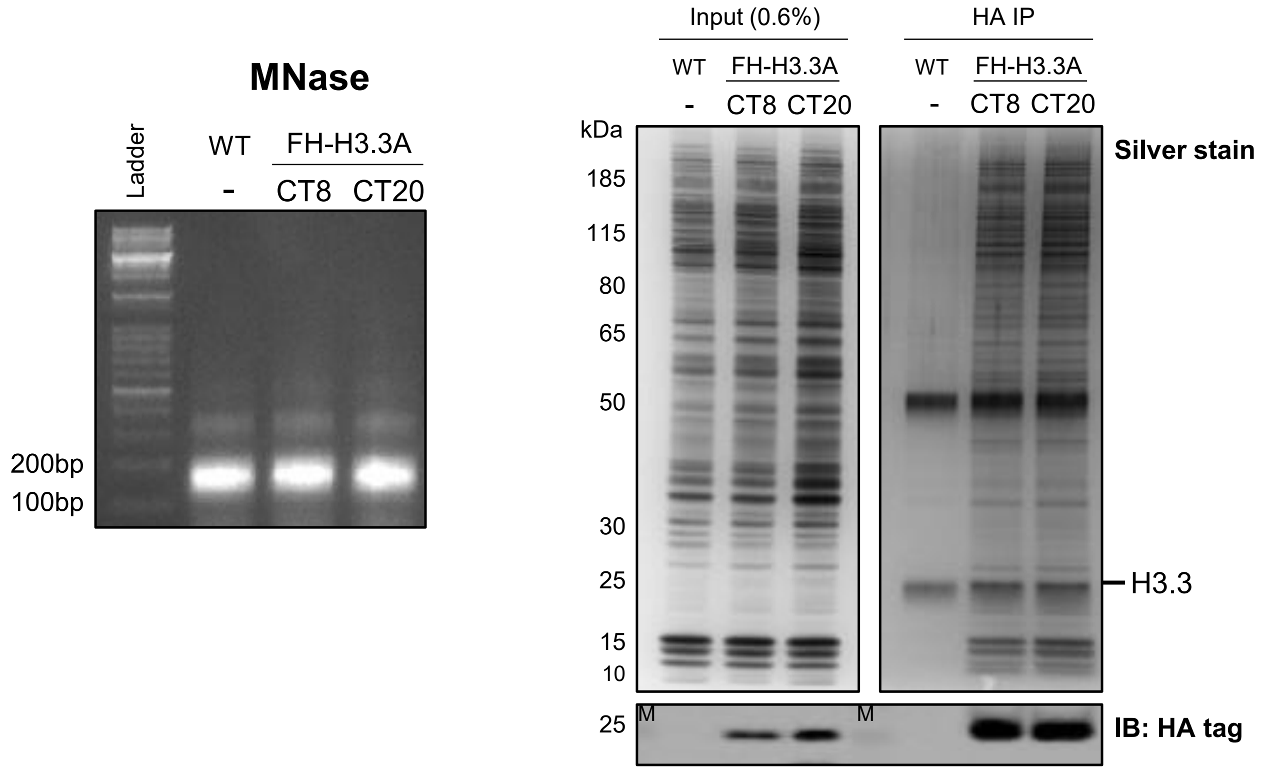

Supplementary Figure 1 *Letkova et al.*

a

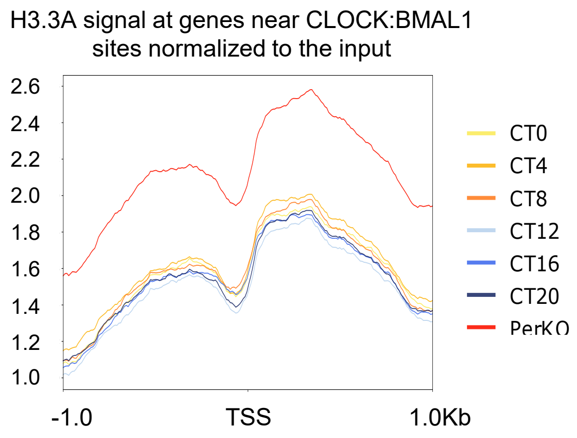

Supplementary Figure 2 *Letkova et al.*

a

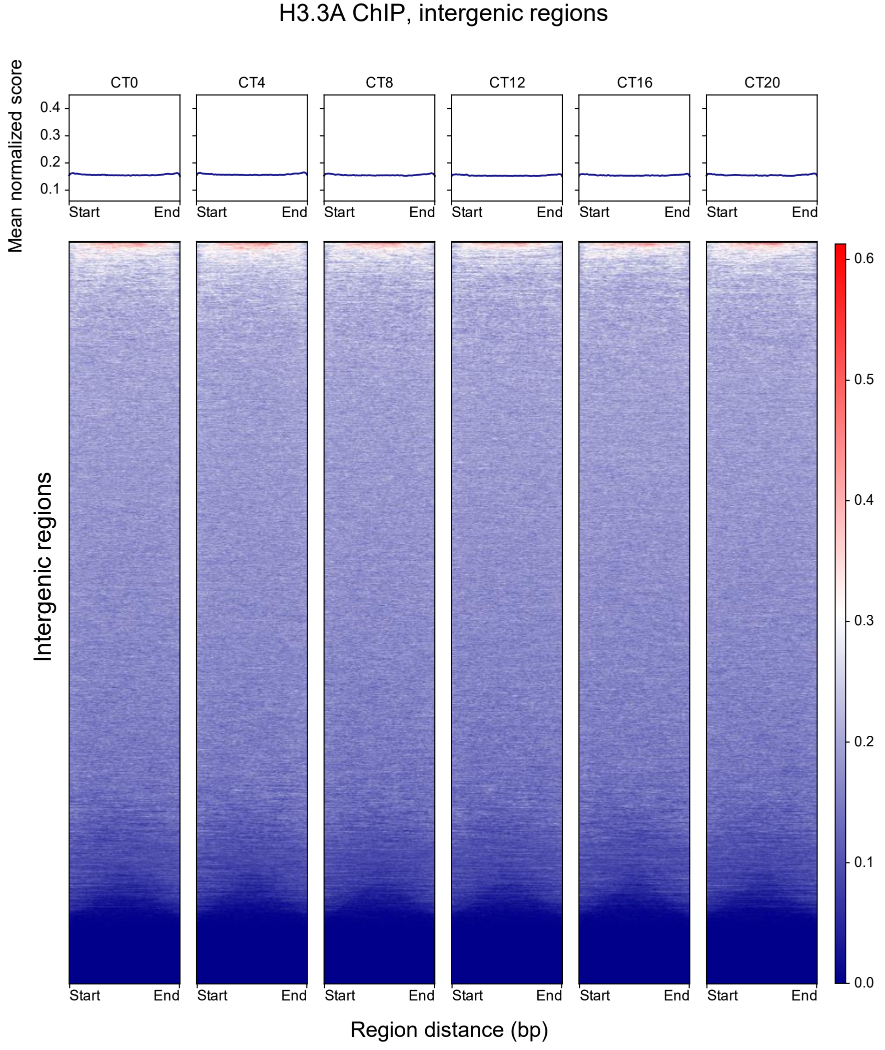

b

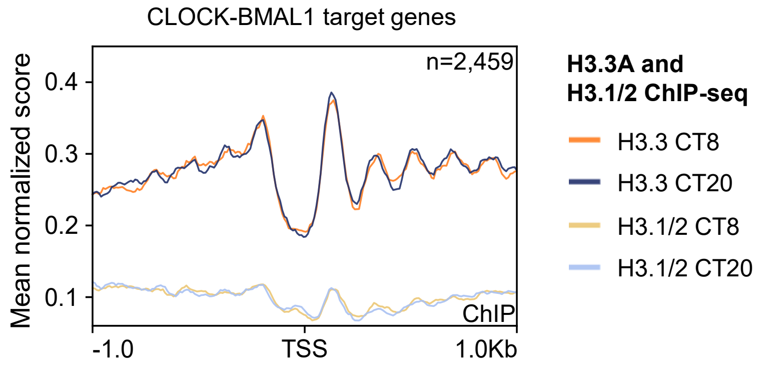

Supplementary Figure 3 *Letkova et al.*

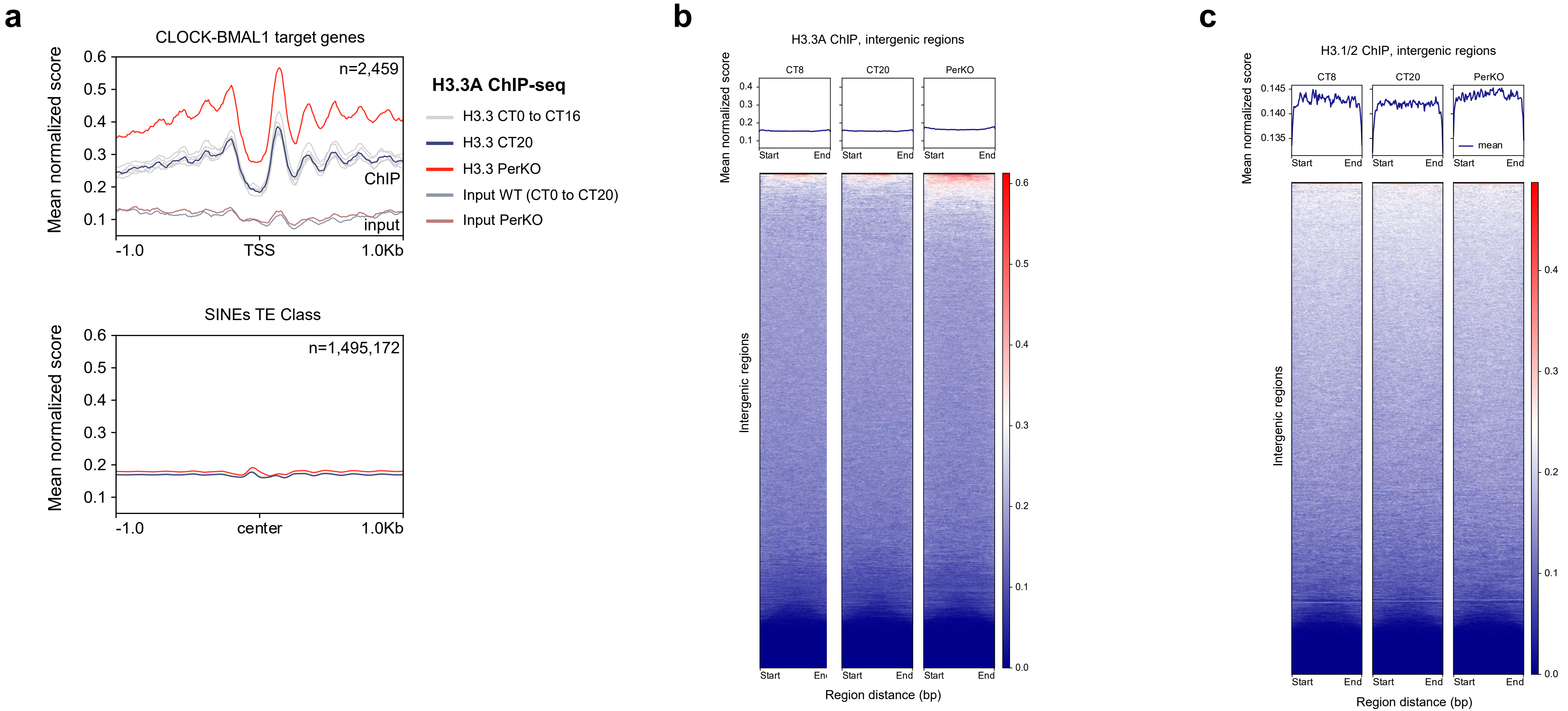

Supplementary Figure 4 *Letkova et al.*

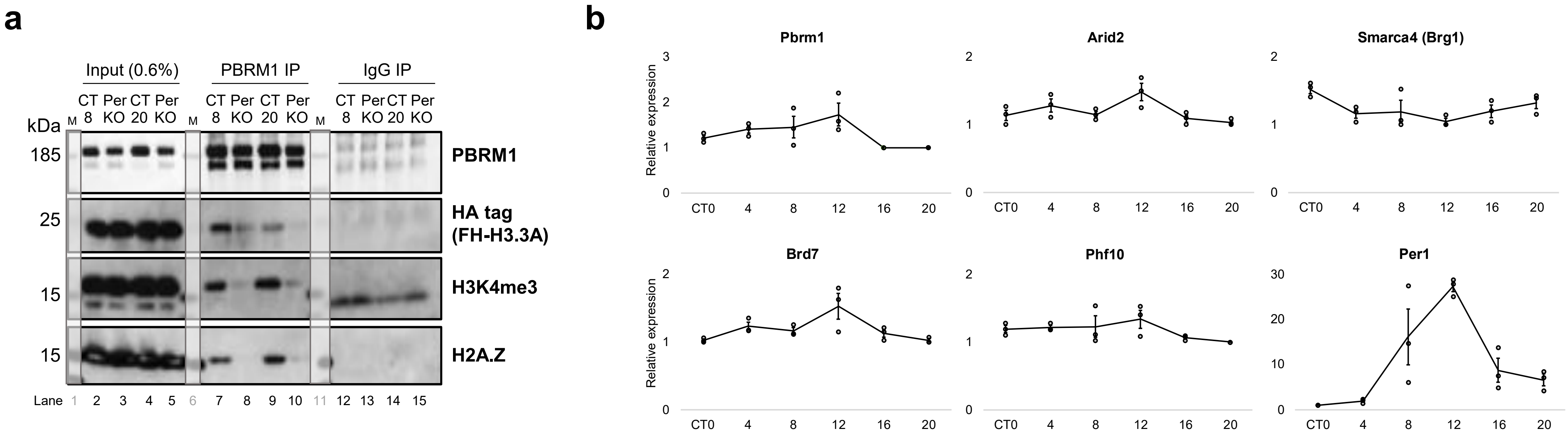

Supplementary Figure 5 *Letkova et al.*

a

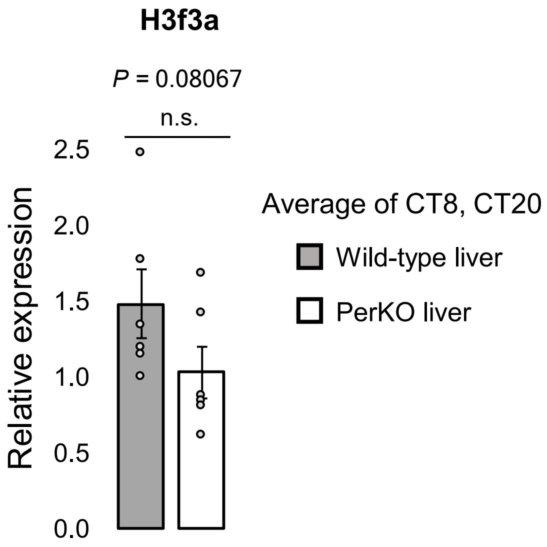

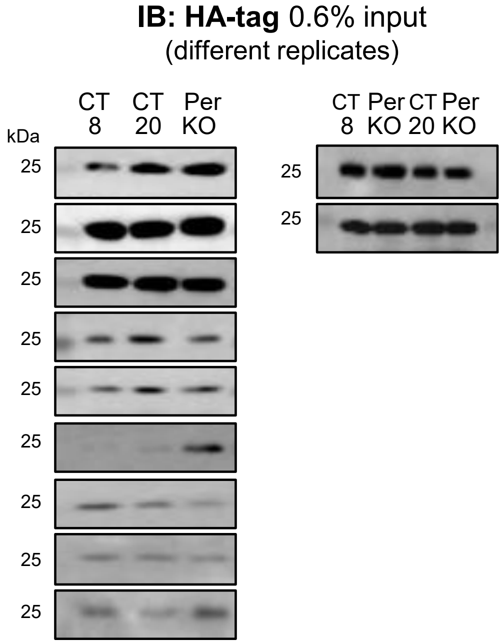

b

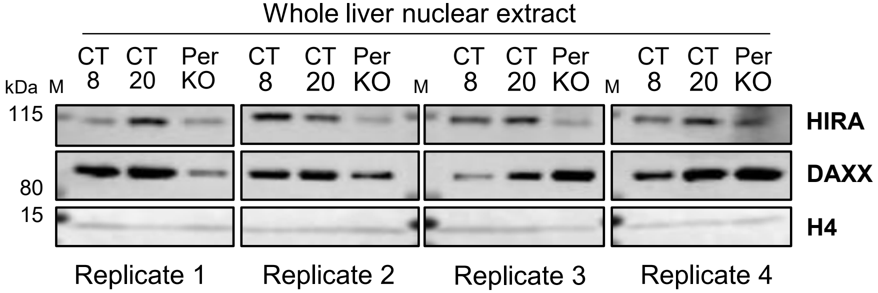

Supplementary Figure 6 *Letkova et al.*

a

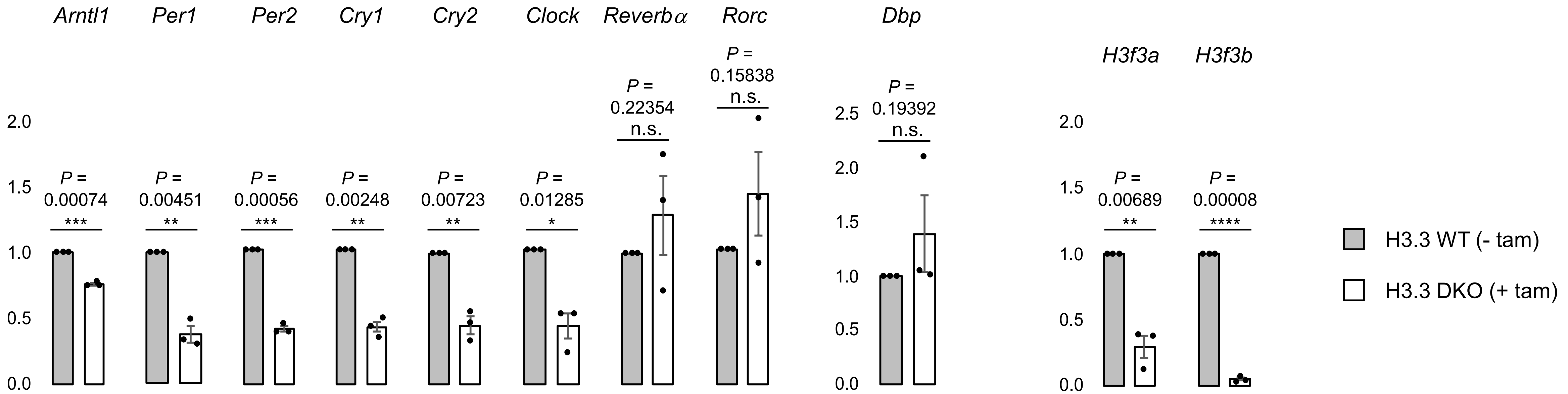

Supplementary Figure 7 *Letkova et al.*

a

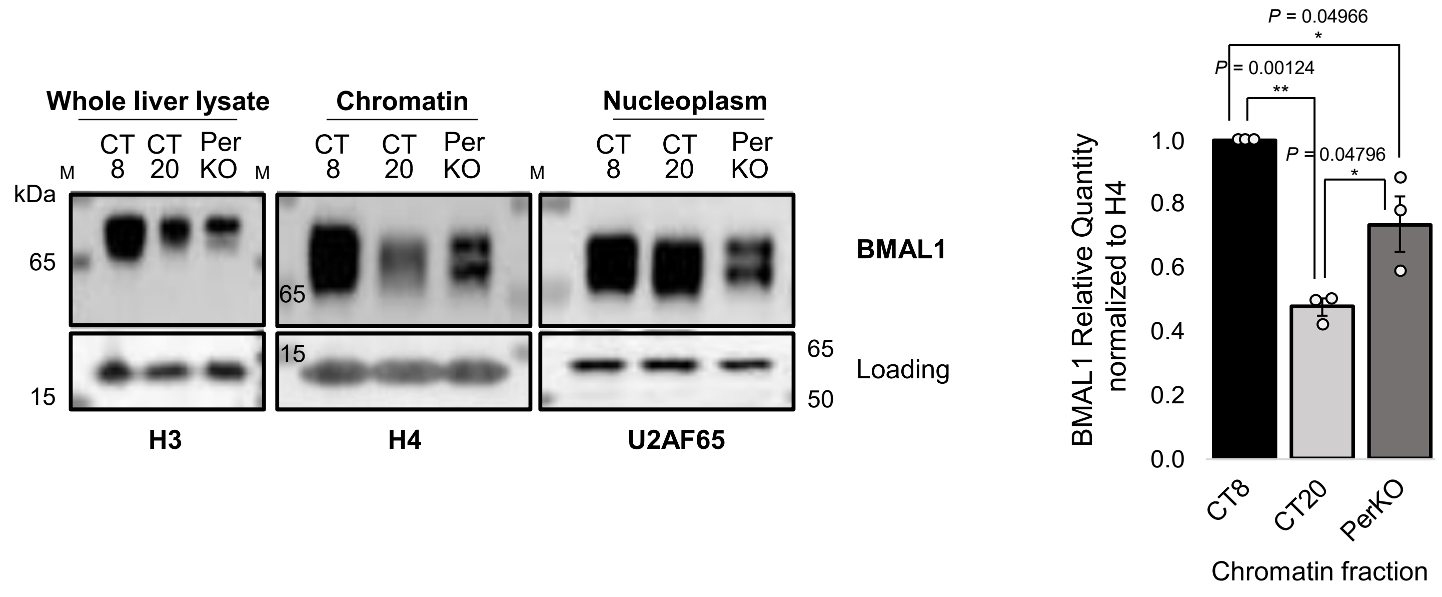

b

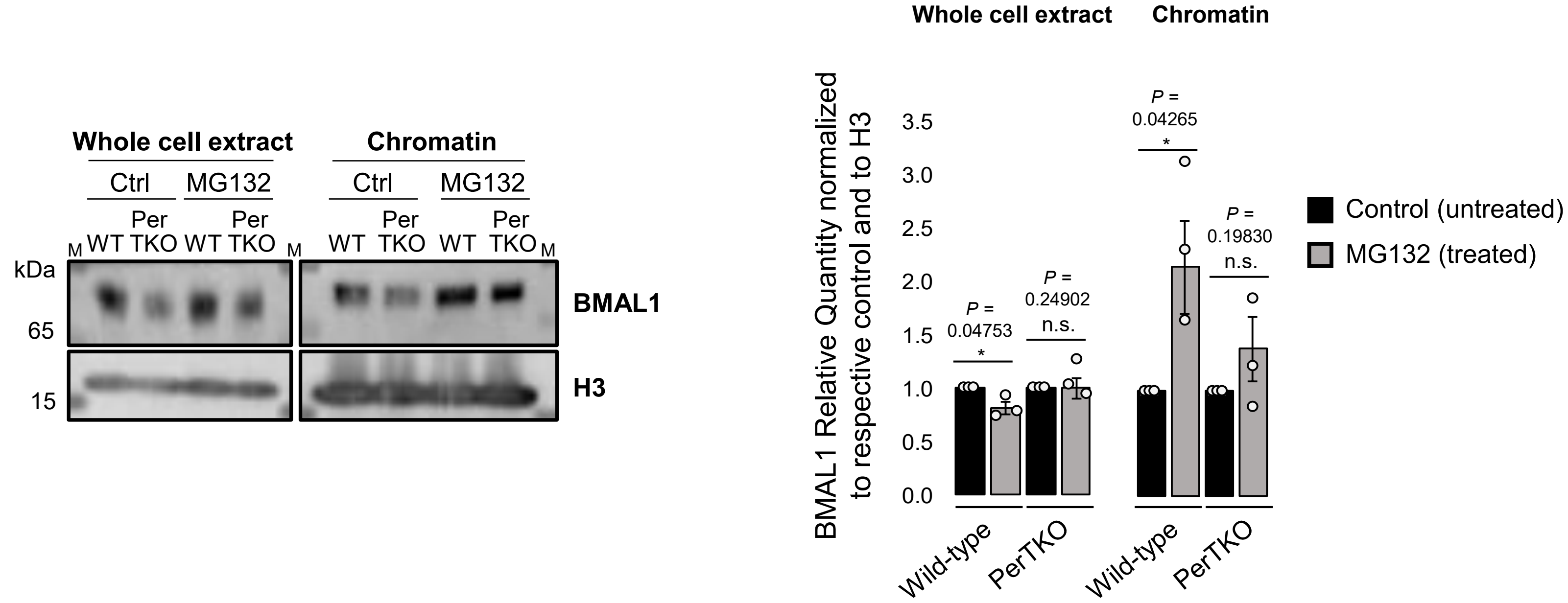

Supplementary Figure 8 *Letkova et al.*

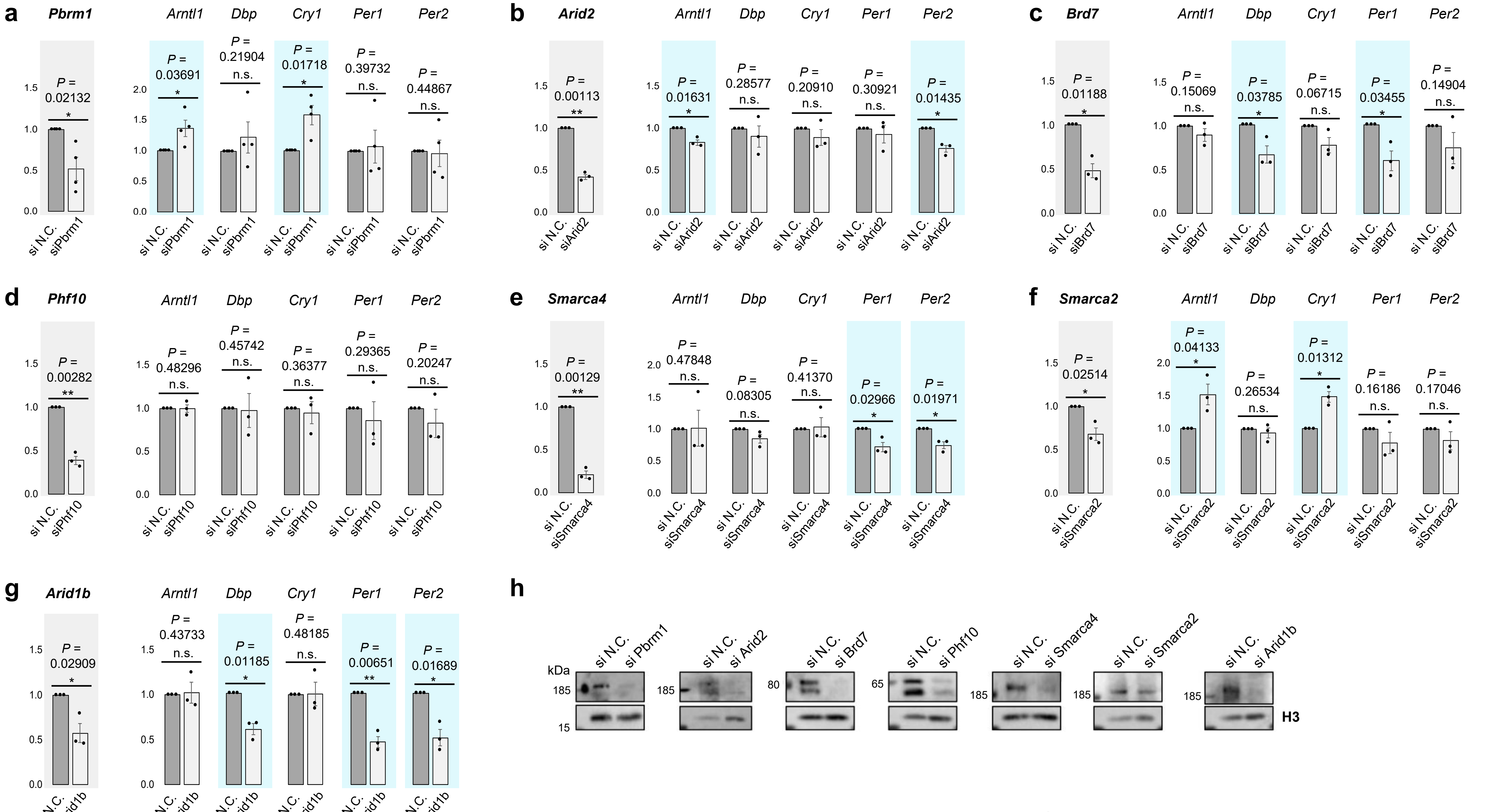

Supplementary Figure 9 *Letkova et al.*

a

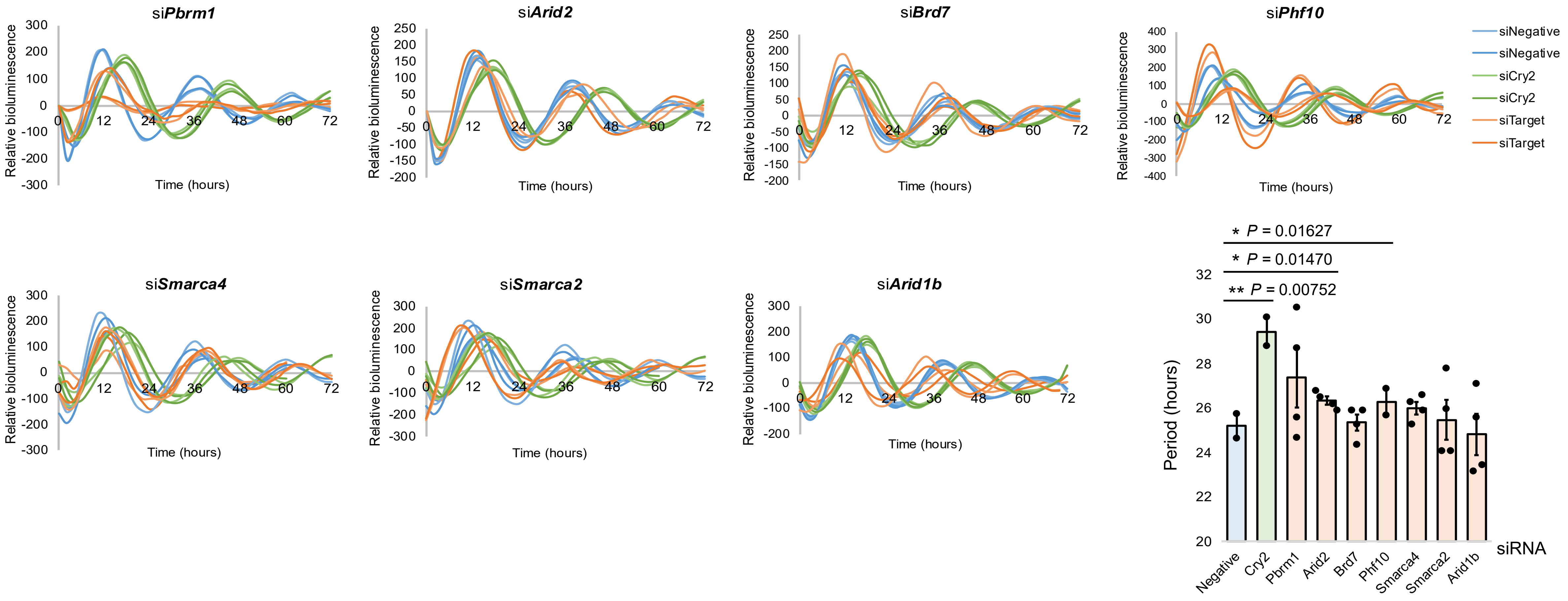

Supplementary Figure 10 *Letkova et al.*

Supplementary Figure Legends

Supplementary Figure 1. Native purification of H3.3A liver protein complexes.

(a) Workflow of native H3.3A purification method from mononucleosome-enriched soluble FH-H3.3A wild-type and PerKO liver chromatin, followed by mass spectrometry. This figure was created in BioRender. Letkova, D. (2025) https://BioRender.com/vjtgp5b

(b) Verification of soluble chromatin fraction enrichment in mononucleosomes on 1.2% agarose gel (left panel), and of the purification method specificity by silver staining, comparing wild-type negative control with FH-H3.3A purifications (right panel). Results are representative of $n > 3$ independent biological replicates.

Supplementary Figure 2. H3.3A signal at genes near CLOCK-BMAL1 binding sites normalized to the input.

(a) H3.3A signal at genes near CLOCK-BMAL1 binding sites at all CT, normalized to the input. Results were obtained from $n = 3$ independent biological replicates, see Supplementary Data 3.

Supplementary Figure 3. H3.3A occupancy dynamics at intergenic regions and CLOCK-BMAL1 target genes.

(a) H3.3A occupancy at intergenic regions. Y-axis corresponds to the mean of normalized scores per genomic regions. X-axis represents the region distance in bp. Heat-map represents an alternative visualization of H3.3A enrichment at intergenic regions. Results were obtained from $n = 3$ independent biological replicates, see Supplementary Data 3.

(b) H3.3A enrichment compared to canonical H3.1/2 at TSS of CLOCK-BMAL1 target genes. Y-axis corresponds to the mean of normalized scores per genomic regions. X-axis represents the distance from the TSS (± 1kb). Each color represents a given CT, with yellow/orange for the day- and blue for the night-time point, as indicated in the figure. Results were obtained from $n = 3$ independent biological replicates, see Supplementary Data 3.

Supplementary Figure 4. H3.3A enrichment on wild-type vs. PerKO chromatin.

(a) H3.3A occupancy at TSS of CLOCK-BMAL1 target genres (upper panel) and SINE repeats (lower panel). Y-axis corresponds to the mean of normalized scores per genomic regions. X-axis represents the distance from the TSS or the center of the given site/region (± 1kb). Highlighted is H3.3A enrichment at CT20 in blue compared to the enrichment in PerKO in red and respective inputs; CT0-CT16 are represented in light grey, as indicated in the figure. Results were obtained from *n* = 3 independent biological replicates, see Supplementary Data 3.

(b, c) H3.3A and H3.1/2 enrichment at intergenic regions. Y-axis corresponds to the mean of normalized scores per genomic regions. X-axis represents the region distance in bp. Heat-map represents an alternative visualization of H3.3A (b) and H3.1/2 (c) enrichment at intergenic regions. Results were obtained from *n* = 3 independent biological replicates, see Supplementary Data 3.

Supplementary Figure 5. Histone marks and variants in PBAF complexes, and expression pattern of subunits.

(a) Native PBRM1 IP at day vs. night time-points in FH-H3.3A and PerKO livers (lanes 7-10); IgG antibody was used as a negative control (lanes 12-15). 0.6% of the nuclear extracts were loaded as Input (lanes 2-5). Immunoblotting of PBRM1, HA (FH-H3.3A), H3K4me3 and H2A.Z. M, protein ladder. Shown are the results from the same IP replicate as in the Figure 3b. Results are representative of *n* = 4 independent biological replicates, see Source data.

(b) mRNA relative expression levels of specific PBAF components and shared ATPase subunit *Brg1* over circadian time, normalized to *Rps9*; *Per1* was used as control. Results are represented as mean ± s.e.m. with individual points indicated for each replicate. Results are representative of *n* = 3 independent biological replicates.

Supplementary Figure 6. Expression of H3.3A and chaperones.

(a) mRNA relative expression levels of *H3f3a* normalized to *Rps9*. Shown is the average of *H3f3a* relative expression at CT8 and CT20 in wild-type and PerKO livers. Results are representative of *n* = 3 independent biological replicates and represented

as mean ± s.e.m. with individual points indicated for each replicate. *P*-value between wild-type and PerKO was calculated with paired, one-tailed t-test; *P-value* = 0.08067, non-significant. In addition, shown are protein levels of H3.3A in input fractions across multiple experiments.

(b) HIRA and DAXX nuclear protein levels at day- vs. night-time points in wild-type and PerKO livers. 5ug of total nuclear proteins were loaded; H4 was used as loading control. Shown are results from *n* = 4 independent biological replicates.

Supplementary Figure 7. Impact of H3.3 loss on clock gene expression.

(a) mRNA relative expression levels of core-clock and clock output genes in non-synchronized H3.3 wild-type (tamoxifen-untreated) and H3.3DKO (tamoxifen-treated) MEFs, normalized to *Rps9*. Results are represented as mean ± s.e.m. with individual points indicated for each replicate. *P*-values were calculated with paired, one-tailed t-test; they are indicated in the figure. Results are representative of *n* = 3 independent biological replicates.

Supplementary Figure 8. BMAL1 presence on chromatin and higher turnover in absence of PER.

(a) BMAL1 protein levels in whole liver lysate, chromatin and nucleoplasm fractions. 15ug of protein were loaded; H3, H4 and U2AF65 were used as loading controls. Bar graph shows the mean ± s.e.m. of BMAL1 relative quantity on chromatin normalized to H4, and setting CT8 as reference at 1, with individual points of normalized ratios indicated for each replicate. *P*-values were calculated with paired, one-tailed t-test. Results are representative of *n* = 3 independent biological replicates.

(b) BMAL1 protein levels in wild-type or PerTKO lung fibroblasts. 3ug of protein were loaded for the whole cell extract and 10ug for chromatin; H3 was used as loading control. BMAL1 levels are shown in untreated (Ctrl) or treaded (MG132) cells with proteasome inhibitor MG132 at 10uM 4h prior extraction. Bar graph shows the mean ± s.e.m. of BMAL1 relative quantity normalized to H3, and to the control samples respective to each condition, with individual points indicated for each replicate. *P*-values were calculated with paired, one-tailed Welch's (unequal variance) t-test. Results are representative of *n* = 3 independent replicates.

Supplementary Figure 9. Impact of specific PBAF and cBAF components on clock gene expression.

(a-g) mRNA relative expression of core-clock and clock output genes upon siRNA-mediated knockdown of specific PBAF-cBAF components in non-synchronized U2OS cell line, normalized to *Rps9*. Results are represented as mean ± s.e.m. with individual points indicated for each replicate. *P*-values were calculated with paired, one-tailed t-test; they are indicated in the figure. Results are representative of *n* = 4 independent biological replicates for *Pbrm1* knockdown and *n* = 3 independent biological replicates for the remaining components.

(h) Verification of the PBAF-cBAF components knockdown efficiency by western-blotting. Shown are the results from pooled samples from all replicates.

Supplementary Figure 10. Reanalysis of published datasets for PBAF and cBAF specific components siRNA screens.

(a) Real-time bioluminescence recordings of Bmal1: Luciferase rhythms is U2OS cell line post- knock-down of specific PBAF and cBAF components, reanalyzed from published datasets of genome-wide siRNA screens [45]. Shown are negative control siRNA (blue), *Cry2* siRNA (green) and specific PBAF or cBAF components as target siRNA (orange). Y-axis represents relative bioluminescence and X-axis the time of recordings in hours. Every available replicate for given siRNA on corresponding plate was taken into account for the analysis. Statistical analysis for period length was performed with available data from the published study [45] (see Methods section for further information). Results are represented as mean ± s.e.m. with individual points indicated for all available replicates. *P*-values were calculated with paired, one-tailed t-test, comparing the negative siRNA to target siRNA values. Significant *P*-values are directly marked on the graph: positive control *Cry2*, ***P* = 0.00752; *Arid2,* **P* = 0.01470; *Phf10,* **P* = 0.01627. Remaining *P*-values were not significant: *Pbrm1, P* = 0.10539; *Brd7, P* = 0.41778; *Smarca 4*, *P* = 0.06671; *Smarca2*, *P* = 0.38670; *Arid1b*, *P* = 0.37158.
