# Supplementary material for: PBAF/cBAF reorganization on H3.3 chromatin regulates BMAL1 activity in the absence of circadian negative feedback
Source: Nat Commun. 2025 Oct 9;16:9000. doi: 10.1038/s41467-025-64045-2 (PMC12511354; doi:10.1038/s41467-025-64045-2)
Supplement: Supplementary file 2 — Description of Additional Supplementary Files [file 41467_2025_64045_MOESM2_ESM.pdf]

**Description of Additional Supplementary Files**

File name: Supplementary Data 1

Description: These data contain the tables related to the mass spectrometry analysis, including the number of peptides and abundance ratios with adjusted p-values for SWI/SNF remodeler components detected in the purified H3.3A complexes. Results are representative of *n* = 2 biological replicates. Abundance ratios with corresponding adjusted p-values for each biological replicate (1) or (2) are represented in individual Excel sheets as follows: H3.3A PerKO night/ H3.3A night ratios (Tables 1a, 1b); H3.3A PerKO night/ H3.3A day ratios (Tables 1c,1d); and H3.3A day/ H3.3A night ratios (Tables 1e,1f). Two-tailed t-test was used for protein quantification, adjusted p-values were obtained by applying the Benjamini-Hochberg procedure.

File name: Supplementary Data 2

Description: These data contain information relative to the quality control analysis of the ChIP samples prior sequencing. The individual peak size (bp) for each replicate, as well as the average peak size (bp) detected at the mononucleosomal level for all ChIP experiments are indicated in the tables. Provided are as well the Tapestation profiles for each replicate and for all ChIP experiments.

File name: Supplementary Data 3

Description: These data contain information relative to the analysis of the ChIP-Seq datasets generated in this study, including the number of raw and down-sampled reads.

File name: Supplementary Data 4

Description: These data contain the list and sequences of all the qPCR primers used in this study, including their origin.
