# Supplementary material for: PBAF/cBAF reorganization on H3.3 chromatin regulates BMAL1 activity in the absence of circadian negative feedback
Source: Nat Commun. 2025 Oct 9;16:9000. doi: 10.1038/s41467-025-64045-2 (PMC12511354; doi:10.1038/s41467-025-64045-2)
Supplement: Supplementary file 4 — Reporting Summary [file 41467_2025_64045_MOESM4_ESM.pdf]

nature portfolio

Corresponding author(s): Kiran PADMANABHAN

Last updated by author(s): Aug 25, 2025

Reporting Summary

Nature Portfolio wishes to improve the reproducibility of the work that we publish. This form provides structure for consistency and transparency in reporting. For further information on Nature Portfolio policies, see our Editorial Policies and the Editorial Policy Checklist.

Statistics

For all statistical analyses, confirm that the following items are present in the figure legend, table legend, main text, or Methods section.

n/a	Confirmed	
☐	☒	The exact sample size (*n*) for each experimental group/condition, given as a discrete number and unit of measurement
☐	☒	A statement on whether measurements were taken from distinct samples or whether the same sample was measured repeatedly
☐	☒	The statistical test(s) used AND whether they are one- or two-sided<br>*Only common tests should be described solely by name; describe more complex techniques in the Methods section.*
☒	☐	A description of all covariates tested
☒	☐	A description of any assumptions or corrections, such as tests of normality and adjustment for multiple comparisons
☐	☒	A full description of the statistical parameters including central tendency (e.g. means) or other basic estimates (e.g. regression coefficient) AND variation (e.g. standard deviation) or associated estimates of uncertainty (e.g. confidence intervals)
☐	☒	For null hypothesis testing, the test statistic (e.g. *F*, *t*, *r*) with confidence intervals, effect sizes, degrees of freedom and *P* value noted<br>*Give P values as exact values whenever suitable.*
☒	☐	For Bayesian analysis, information on the choice of priors and Markov chain Monte Carlo settings
☒	☐	For hierarchical and complex designs, identification of the appropriate level for tests and full reporting of outcomes
☒	☐	Estimates of effect sizes (e.g. Cohen's *d*, Pearson's *r*), indicating how they were calculated

Our web collection on statistics for biologists contains articles on many of the points above.

Software and code

Policy information about availability of computer code

Data collection	No software was used for data collection.
Data analysis	All the scripts for ChIP-Seq analysis are available at: https://github.com/Thrylia/ChIP_HA-Complexe [https://doi.org/10.5281/zenodo.16793659]

For manuscripts utilizing custom algorithms or software that are central to the research but not yet described in published literature, software must be made available to editors and reviewers. We strongly encourage code deposition in a community repository (e.g. GitHub). See the Nature Portfolio guidelines for submitting code & software for further information.

Data

Policy information about availability of data

All manuscripts must include a data availability statement. This statement should provide the following information, where applicable:

- Accession codes, unique identifiers, or web links for publicly available datasets
- A description of any restrictions on data availability
- For clinical datasets or third party data, please ensure that the statement adheres to our policy

ChIP-Seq data can be obtained in the GEO database under accession code GSE284165, at https://www.ncbi.nlm.nih.gov/geo/query/acc.cgi?acc=GSE284165.

Proteomics Data are available via ProteomeXchange with identifier PXD061974 and 10.6019/PXD061974, at https://www.ebi.ac.uk/pride/archive/projects/PXD061974

Research involving human participants, their data, or biological material

Policy information about studies with human participants or human data. See also policy information about sex, gender (identity/presentation), and sexual orientation and race, ethnicity and racism.

Reporting on sex and gender	n/a
Reporting on race, ethnicity, or other socially relevant groupings	n/a
Population characteristics	n/a
Recruitment	n/a
Ethics oversight	n/a

Note that full information on the approval of the study protocol must also be provided in the manuscript.

Field-specific reporting

Please select the one below that is the best fit for your research. If you are not sure, read the appropriate sections before making your selection.

☒ Life sciences ☐ Behavioural & social sciences ☐ Ecological, evolutionary & environmental sciences

For a reference copy of the document with all sections, see nature.com/documents/nr-reporting-summary-flat.pdf

Life sciences study design

All studies must disclose on these points even when the disclosure is negative.

Sample size	The sample sizes are stated in the figure legends. No statistical methods were used to predetermine the sample sizes. The sample sizes are as those typical used in this filed.
Data exclusions	No data were excluded from the analysis.
Replication	Experiments were independently repeated, and only confirmed results are presented. The numbers of replicates are indicated in the figure legends. An Uncropped_membranes_all_replicates_Letkova_et_al file contains all replicates performed for all biochemical experiments in main and supplemental figures.
Randomization	Mouse littermates are indinstiquishable. They were taken randomly for sacrifice at the different time points. Otherwise randomization was not relevant to the study as proper controls were used and both the controls and the treatments samples are prepared in the same manner.
Blinding	Researchers were not blinded during experiments and data analysis, because proper controls were used.

Reporting for specific materials, systems and methods

We require information from authors about some types of materials, experimental systems and methods used in many studies. Here, indicate whether each material, system or method listed is relevant to your study. If you are not sure if a list item applies to your research, read the appropriate section before selecting a response.

Materials & experimental systems

n/a	Involved in the study	
☐	☒	Antibodies
☐	☒	Eukaryotic cell lines
☒	☐	Palaeontology and archaeology
☐	☒	Animals and other organisms
☒	☐	Clinical data
☒	☐	Dual use research of concern
☒	☐	Plants

Methods

n/a	Involved in the study	
☐	☒	ChIP-seq
☐	☐	Flow cytometry
☐	☐	MRI-based neuroimaging

Antibodies

Antibodies used	HA-Tag (C29F4) Rabbit mAb (Cell Signaling, #3724), 1:1000<br>PBRM1/BAF180 (E9X2Z) Rabbit mAb (Cell Signaling, #89123), 1:1000<br>ARID2 (GT7311) Mouse mAb (Sigma-Aldrich, SAB2702340), 1:500<br>ARID2 (GT7311) Mouse mAb (GENETEX, GTX632011), 1:500<br>BRG1/SMARCA4 Rabbit pAb (Bethyl Laboratories, A300-813A), 1:500<br>BRD7 Rabbit pAb (Proteintech, 51009-2-AP), 1:500<br>PHF10 Mouse mAb (Proteintech, 66341-1-Ig), 1:500<br>PHF10 Rabbit pAb (GENETEX, GTX116314), 1:500<br>BMAL1 Rabbit pAb (Bethyl Laboratories, A302-616A), 1:1000<br>CLOCK Mouse mAb (MBL, D334-3), 1:500<br>Histone H3.1/3.2 (1D4F2) Mouse mAb (Active Motif, 61629), 1:500<br>Tri-Methyl-Histone H3 (Lys4) Rabbit pAb (Cell Signaling, #9727), 1:500<br>Acetyl-Histone H3 (Lys115) Rabbit pAb (PTM Bio, PTM-170), 1:1000<br>Histone H3 (acetyl K122) Rabbit pAb (Abcam, ab33309), 1:500<br>Histone H2A.Z Rabbit pAb (Active Motif, 39113), 1:1000<br>Homemade H2A.Z Rabbit pAb (kind gift from Stefan Dimitrov), 1:500<br>ARID1B Rabbit pAb (GENETEX, GTX130708), 1:500<br>SMARCA2 (HL1115) Rabbit mAb (GENETEX, GTX636330), 1:500<br>Histone H3 Rabbit pAb (Proteintech, 17168-1-AP), 1:2000<br>Histone H4 Rabbit pAb (Proteintech, 16047-1-AP), 1:2000<br>U2AF65 Rabbit mAb (Abcam, ab197031), 1:500<br>HIRA (clone WC119) Mouse mAb (Sigma-Aldrich, 04-1488), 1:500<br>DAXX anti-Mouse (Precision antibody), 1:500<br><br>2 - 4.5ug were used for related antibodies used for the immunoprecipitation and ChIP-seq experiments as indicated in the Methods section.
Validation	All antibodies used were validated by the manufacturers (product reference indicated) and when feasible validated in house (H2A.Z antibody for example was generated in-house (kind gift from Stefan Dimitrov, UGA) using conditional knockout cells: PMID:35606517 .

Eukaryotic cell lines

Policy information about cell lines and Sex and Gender in Research

Cell line source(s)	H33 floxed mouse embryonic fibroblasts<br>WT lung fibroblasts: generated in house from mice<br>PerTKO lung fibroblasts: gift from Shin Yamazaki (UT Southwestern)<br>in all cases, cells were generated from pooled tissues from multiple animals.
Authentication	WT lung fibroblasts and H33 floxed mouse embryonic fibroblasts were genereated in house. Floxed alleles were validated by genotyping PCRs as in PMID:23315948 and 35766398. The Per knockout cells were derived from the knockout mouse model generated by David Weaver and Steve Reppert (UMass Medical School) validated by qPCR using primers for each of the three Per isoforms upon receipt.
Mycoplasma contamination	Cell lines are tested for mycoplasma prior to use. If a cell line became contaminated, cells were discarded immediately.
Commonly misidentified lines<br>(See ICLAC register)	n/a

Animals and other research organisms

Policy information about studies involving animals; ARRIVE guidelines recommended for reporting animal research, and Sex and Gender in Research

Laboratory animals	Mus musculus (C57BL/6J); male 8-12 weeks were obtained from Charles River Laboratories. H3.3A males and H3.3A;PerKO males were 10-17 weeks old were raised and housed at the IGFL PEHR animal facility.
Wild animals	This study did not include wild animals.
Reporting on sex	All experiments were performed on male mice. This was done a) to avoid the impact of circulating hormones on biological rhythms and b) to be consistent with 20 +years of literature in the circadian field to be able to take advantage of the large data resources in the community.
Field-collected samples	This study did not include field-collected samples.
Ethics oversight	Animal procedures were carried out according to the French Guidelines for care and use of experimental animals and approved by the ethical committee of the ENS and Univ Lyon (CECAPP), the IGFL's Society for Animal Welfare (SBEA) and the French ministry of Science under APAFIS#5212-2016032914413553 v2.

Note that full information on the approval of the study protocol must also be provided in the manuscript.

Plants

Seed stocks	n/a
Novel plant genotypes	n/a
Authentication	n/a

ChIP-seq

Data deposition

☒ Confirm that both raw and final processed data have been deposited in a public database such as GEO.

☐ Confirm that you have deposited or provided access to graph files (e.g. BED files) for the called peaks.

Data access links
May remain private before publication.

https://www.ncbi.nlm.nih.gov/geo/query/acc.cgi?acc=GSE284165

Files in database submission

GSM8679148 H3.3A circadian ChIP (wild-type, CT0_replicate 1)
GSM8679149 H3.3A circadian ChIP (wild-type, CT0_replicate 2)
GSM8679150 H3.3A circadian ChIP (wild-type, CT0_replicate 3)
GSM8679151 H3.3A circadian ChIP (wild-type, CT4_replicate 1)
GSM8679152 H3.3A circadian ChIP (wild-type, CT4_replicate 2)
GSM8679153 H3.3A circadian ChIP (wild-type, CT4_replicate 3)
GSM8679154 H3.3A circadian ChIP (wild-type, CT8_replicate 1)
GSM8679155 H3.3A circadian ChIP (wild-type, CT8_replicate 2)
GSM8679156 H3.3A circadian ChIP (wild-type, CT8_replicate 3)
GSM8679157 H3.3A circadian ChIP (wild-type, CT12_replicate 1)
GSM8679158 H3.3A circadian ChIP (wild-type, CT12_replicate 2)
GSM8679159 H3.3A circadian ChIP (wild-type, CT12_replicate 3)
GSM8679160 H3.3A circadian ChIP (wild-type, CT16_replicate 1)
GSM8679161 H3.3A circadian ChIP (wild-type, CT16_replicate 2)
GSM8679162 H3.3A circadian ChIP (wild-type, CT16_replicate 3)
GSM8679163 H3.3A circadian ChIP (wild-type, CT20_replicate 1)
GSM8679164 H3.3A circadian ChIP (wild-type, CT20_replicate 2)
GSM8679165 H3.3A circadian ChIP (wild-type, CT20_replicate 3)
GSM8679166 H3.3A circadian ChIP (PerKO, CT20_replicate 1)
GSM8679167 H3.3A circadian ChIP (PerKO, CT20_replicate 2)
GSM8679168 H3.3A circadian ChIP (PerKO, CT20_replicate 3)
GSM8679169 H3.3A PerKO CT20 input_replicate1
GSM8679170 H3.3A PerKO CT20 input_replicate3
GSM8679171 H3.3A circadian input (all CTs pooled)_replicate1
GSM8679172 H3.3A circadian input (all CTs pooled)_replicate3
GSM8679173 H3.1/H3.2 ChIP (wild-type, CT8_replicate1)
GSM8679174 H3.1/H3.2 ChIP (wild-type, CT8_replicate2)
GSM8679175 H3.1/H3.2 ChIP (wild-type, CT8_replicate3)
GSM8679176 H3.1/H3.2 ChIP (wild-type, CT20_replicate1)
GSM8679177 H3.1/H3.2 ChIP (wild-type, CT20_replicate2)
GSM8679178 H3.1/H3.2 ChIP (wild-type, CT20_replicate3)
GSM8679179 H3.1/H3.2 ChIP (PerKO, CT20_replicate1)
GSM8679180 H3.1/H3.2 ChIP (PerKO, CT20_replicate2)
GSM8679181 H3.1/H3.2 ChIP (PerKO, CT20_replicate3)
GSM8679182 BMAL1 ChIP (wild-type, CT8_replicate1)
GSM8679183 BMAL1 ChIP (wild-type, CT8_replicate2)
GSM8679184 BMAL1 ChIP (wild-type, CT8_replicate3)
GSM8679185 BMAL1 ChIP (wild-type, CT20_replicate1)
GSM8679186 BMAL1 ChIP (wild-type, CT20_replicate2)
GSM8679187 BMAL1 ChIP (wild-type, CT20_replicate3)
GSM8679188 BMAL1 ChIP (PerKO, CT20_replicate1)
GSM8679189 BMAL1 ChIP (PerKO, CT20_replicate2)
GSM8679190 BMAL1 ChIP (PerKO, CT20_replicate3)
GSM8679191 H3.1/H3.2/BMAL1 ChIP PerKO CT20 input_replicate1
GSM8679192 H3.1/H3.2/BMAL1 ChIP PerKO CT20 input_replicate3

GSM8679193 H3.1/H3.2/BMAL1 ChIP input (all CTs pooled)_replicate1
GSM8679194 H3.1/H3.2/BMAL1 ChIP input (all CTs pooled)_replicate3

Genome browser session (e.g. UCSC)

Provide a link to an anonymized genome browser session for "Initial submission" and "Revised version" documents only, to enable peer review. Write "no longer applicable" for "Final submission" documents.

Methodology

Replicates

3 replicates for each circadian time point (CT). ChIPs were performed for H3.3, H3.1/2 and BMAL1 at various CTs as indicated.

Sequencing depth

Each replicate had between 30 and 54 million raw reads, 25-44 million uniquely mapped reads and were sequenced PE150.

Antibodies

Histone H3.1/3.2 (1D4F2) Mouse mAb
for HA-H3.3, HA-Tag (C29F4) Rabbit mAb (Cell Signaling, #3724)
BMAL1 Rabbit pAb (Bethyl Laboratories, A302-616A

Peak calling parameters

The alignment part was done with Bowtie2 5.5.4 with the following command line :

```
bowtie2 \
   -p $SLURM_CPUS_PER_TASK  \
   --very-sensitive \
   --phred33 \
   --no-mixed \
   --no-discordant \
   --dovetail \
   -x $genome_mm39.index \
   -1 $reads1_trim -2 $reads2_trim \
   -S $replicate_name.sam
```

In order to compare the alignments obtained for each sample and represent them, deepTools 3.5.5 54 was selected to create a matrix of intensity scores (computeMatrix) from the normalized alignments (bamcoverage). These were then plotted either as a heatmap (plotHeatmap) or as an average profile plot (plotProfile):

```
bamCoverage \
   -b $sample_name.sort.bam \
   --outFileFormat bigwig \
   --outFileName $sample_name.rpgc.bw \
   --normalizeUsing RPGC \
   --effectiveGenomeSize 2495461690 \
   --MNase \
   --numberOfProcessors $SLURM_CPUS_PER_TASK \
   --minFragmentLength 80 \
   --maxFragmentLength 200 \
   --smoothLength 80
computeMatrix reference-point \
   -S $sample_name.rpgc.bw \
   -R $region_to_check \
   --beforeRegionStartLength 1000 \
   --afterRegionStartLength 1000 \
   --referencePoint center \
   --sortRegions keep \
   --numberOfProcessors $SLURM_CPUS_PER_TASK \
   --smartLabels \
   --metagene \
   --outFileName $name_output.$region_to_check.bw.gz
   # --referencePoint center or TSS : depends the region to check
plotProfile -m $name_output.$region_to_check.bw.gz \
   -o $name_output.$region_to_check.profile.svg \
   --plotFileFormat svg \
   --perGroup \
   --plotType lines \
   --numPlotsPerRow 1 \
   --plotHeight 9 \
   --plotWidth 7 \
   --samplesLabel Label1 Label2 Label3 ... \
   --legendLocation best \
   --plotTitle "Title"
```

Data quality

FastQC, MultiQC: Reads quality
samtools flagstat, samtools stats: %reads Unique Mapped, %reads Duplicated, etc. See supplementary table.
IGV, samtools-depth : signal distribution

Software

bedtools/2.30.0
bowtie2/2.5.4

cutadapt/4.5
deeptools/3.5.4
fastqc/0.12.1
homer/4.11
multiqc/1.13
picard/2.23.5
samtools/1.18

Flow Cytometry

Plots

Confirm that:

- ☐ The axis labels state the marker and fluorochrome used (e.g. CD4-FITC).
- ☐ The axis scales are clearly visible. Include numbers along axes only for bottom left plot of group (a 'group' is an analysis of identical markers).
- ☐ All plots are contour plots with outliers or pseudocolor plots.
- ☐ A numerical value for number of cells or percentage (with statistics) is provided.

Methodology

Sample preparation	*Describe the sample preparation, detailing the biological source of the cells and any tissue processing steps used.*
Instrument	*Identify the instrument used for data collection, specifying make and model number.*
Software	*Describe the software used to collect and analyze the flow cytometry data. For custom code that has been deposited into a community repository, provide accession details.*
Cell population abundance	*Describe the abundance of the relevant cell populations within post-sort fractions, providing details on the purity of the samples and how it was determined.*
Gating strategy	*Describe the gating strategy used for all relevant experiments, specifying the preliminary FSC/SSC gates of the starting cell population, indicating where boundaries between "positive" and "negative" staining cell populations are defined.*

☐ Tick this box to confirm that a figure exemplifying the gating strategy is provided in the Supplementary Information.

Magnetic resonance imaging

Experimental design

Design type	*Indicate task or resting state; event-related or block design.*
Design specifications	*Specify the number of blocks, trials or experimental units per session and/or subject, and specify the length of each trial or block (if trials are blocked) and interval between trials.*
Behavioral performance measures	*State number and/or type of variables recorded (e.g. correct button press, response time) and what statistics were used to establish that the subjects were performing the task as expected (e.g. mean, range, and/or standard deviation across subjects).*

Acquisition

Imaging type(s)	*Specify: functional, structural, diffusion, perfusion.*
Field strength	*Specify in Tesla*
Sequence & imaging parameters	*Specify the pulse sequence type (gradient echo, spin echo, etc.), imaging type (EPI, spiral, etc.), field of view, matrix size, slice thickness, orientation and TE/TR/flip angle.*
Area of acquisition	*State whether a whole brain scan was used OR define the area of acquisition, describing how the region was determined.*

Diffusion MRI ☐ Used ☐ Not used

Preprocessing

Preprocessing software	*Provide detail on software version and revision number and on specific parameters (model/functions, brain extraction, segmentation, smoothing kernel size, etc.).*
Normalization	*If data were normalized/standardized, describe the approach(es): specify linear or non-linear and define image types used for*

Normalization	*transformation OR indicate that data were not normalized and explain rationale for lack of normalization.*
Normalization template	*Describe the template used for normalization/transformation, specifying subject space or group standardized space (e.g. original Talairach, MNI305, ICBM152) OR indicate that the data were not normalized.*
Noise and artifact removal	*Describe your procedure(s) for artifact and structured noise removal, specifying motion parameters, tissue signals and physiological signals (heart rate, respiration).*
Volume censoring	*Define your software and/or method and criteria for volume censoring, and state the extent of such censoring.*

Statistical modeling & inference

Model type and settings	*Specify type (mass univariate, multivariate, RSA, predictive, etc.) and describe essential details of the model at the first and second levels (e.g. fixed, random or mixed effects; drift or auto-correlation).*
Effect(s) tested	*Define precise effect in terms of the task or stimulus conditions instead of psychological concepts and indicate whether ANOVA or factorial designs were used.*

Specify type of analysis: ☐ Whole brain ☐ ROI-based ☐ Both

Statistic type for inference<br>(See Eklund et al. 2016)	*Specify voxel-wise or cluster-wise and report all relevant parameters for cluster-wise methods.*
Correction	*Describe the type of correction and how it is obtained for multiple comparisons (e.g. FWE, FDR, permutation or Monte Carlo).*

Models & analysis

n/a	Involved in the study
☐	☐ Functional and/or effective connectivity
☐	☐ Graph analysis
☐	☐ Multivariate modeling or predictive analysis

Functional and/or effective connectivity	*Report the measures of dependence used and the model details (e.g. Pearson correlation, partial correlation, mutual information).*
Graph analysis	*Report the dependent variable and connectivity measure, specifying weighted graph or binarized graph, subject- or group-level, and the global and/or node summaries used (e.g. clustering coefficient, efficiency, etc.).*
Multivariate modeling and predictive analysis	*Specify independent variables, features extraction and dimension reduction, model, training and evaluation metrics.*
