# Supplementary material for: PBAF/cBAF reorganization on H3.3 chromatin regulates BMAL1 activity in the absence of circadian negative feedback
Source: Nat Commun. 2025 Oct 9;16:9000. doi: 10.1038/s41467-025-64045-2 (PMC12511354; doi:10.1038/s41467-025-64045-2)
Supplement: Supplementary file 6 — Source Data [file 41467_2025_64045_MOESM6_ESM.zip › Source_Data_files_Letkova_et_al/Quantifications_western_blotting_graphs_Letkova_et_al.pdf]

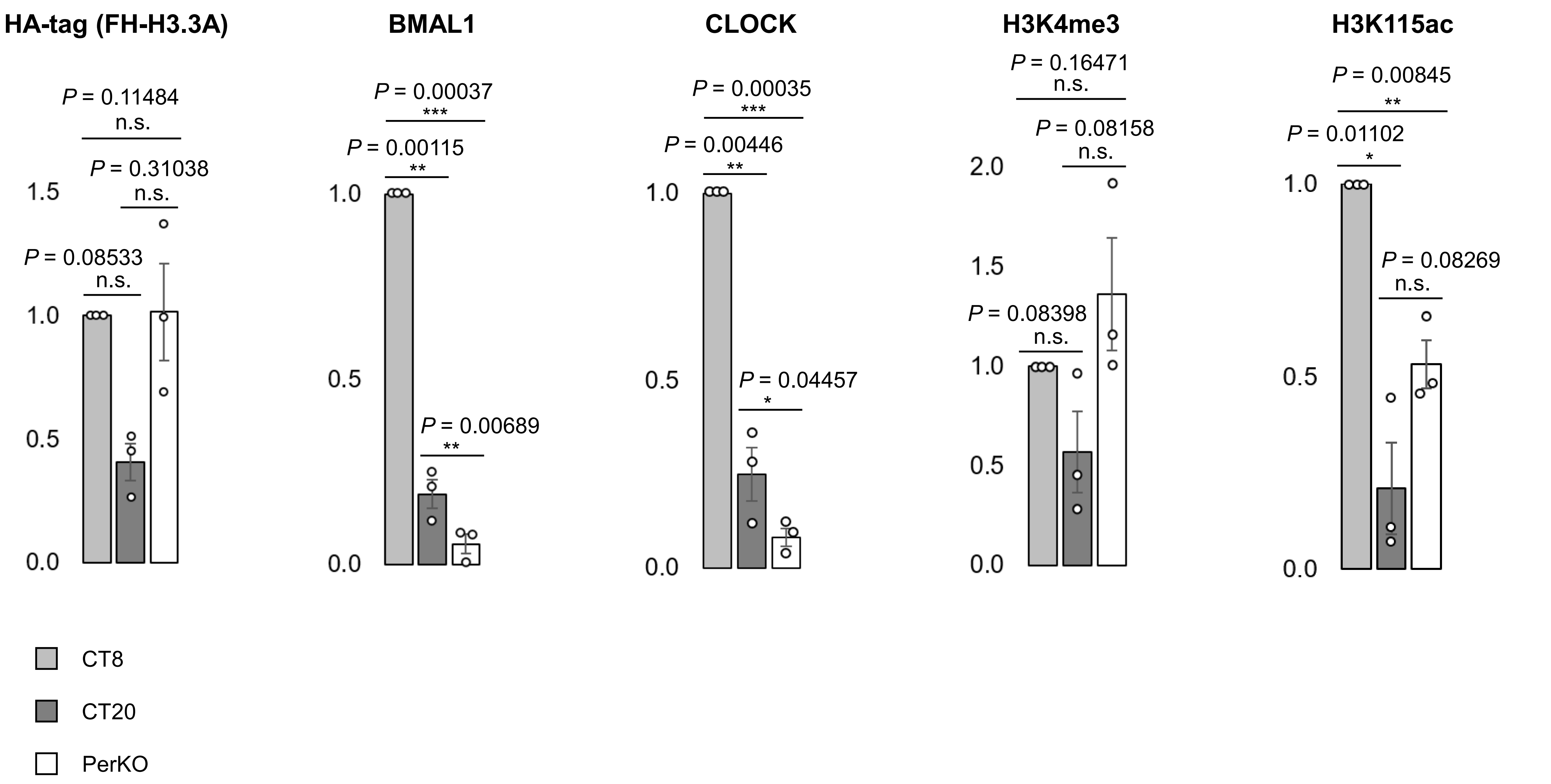

Figures 1a, 5a, (3a for HA-tag)

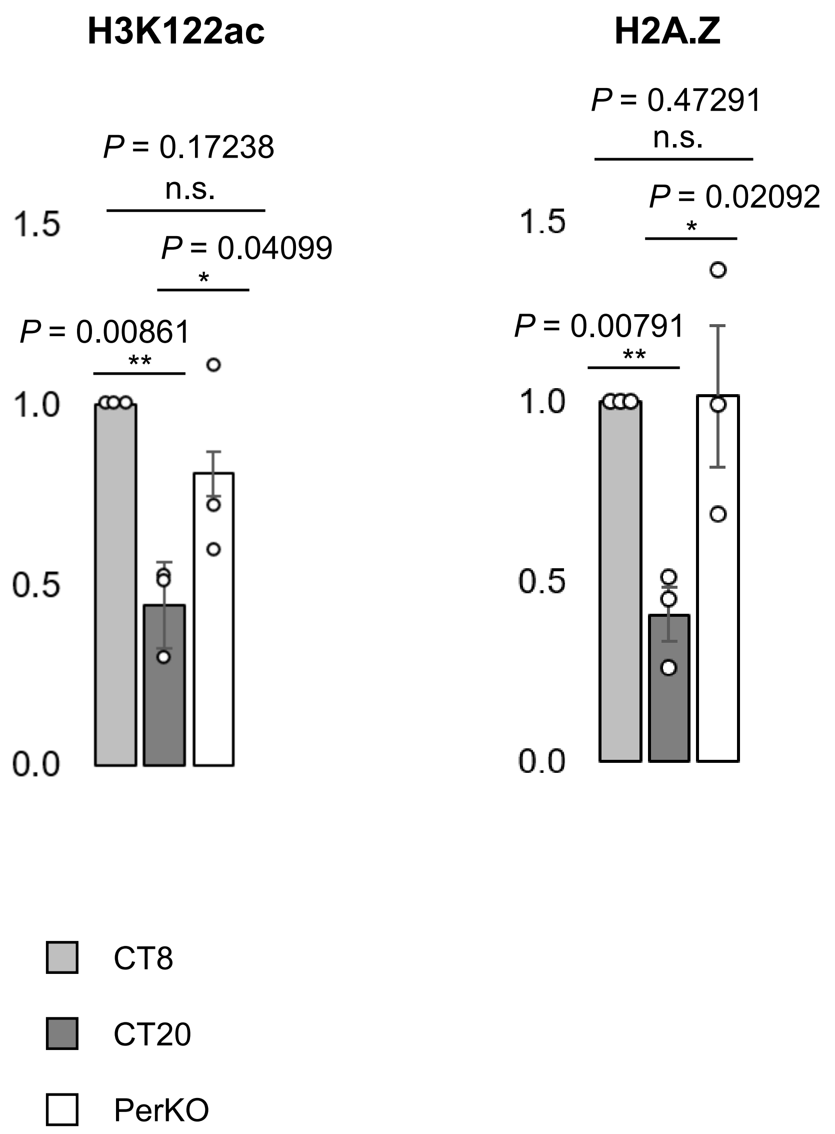

Figures 1a, 5a, (3a for HA-tag)

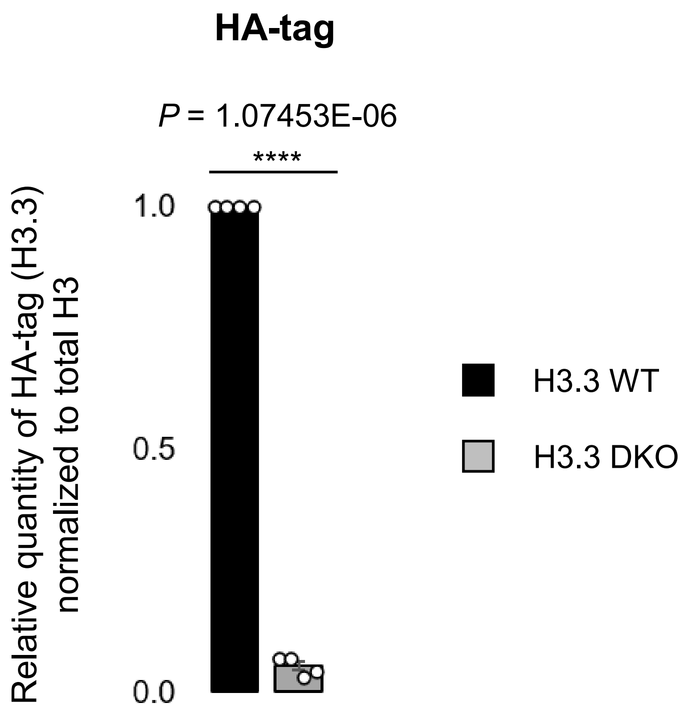
HA-tag
P = 1.07453E-06
****
Relative quantity of HA-tag (H3.3) normalized to total H3
1.0
0.5
0.0
H3.3 WT
H3.3 DKO

Figure 1f

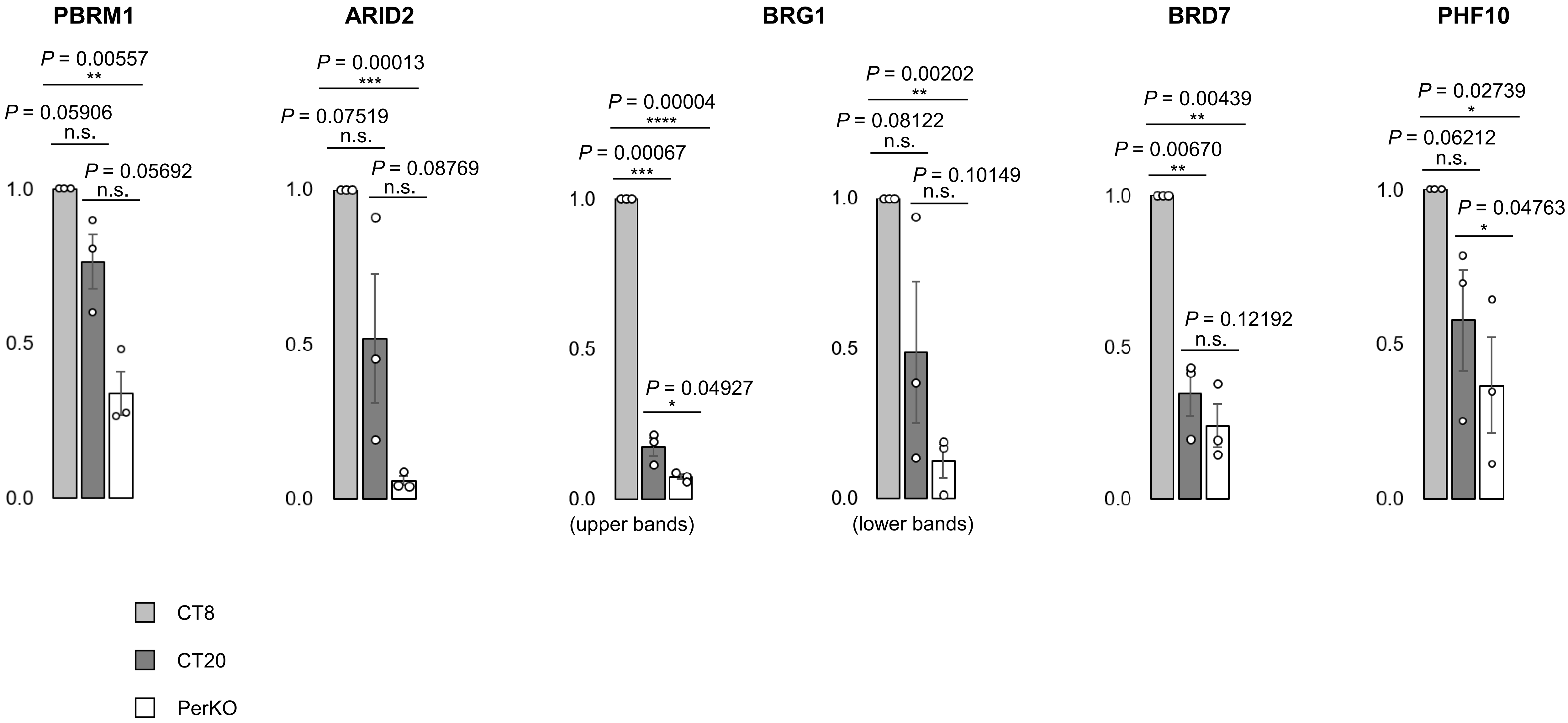
PBRM1
P = 0.00557
**
P = 0.05906
n.s.
P = 0.05692
n.s.
ARID2
P = 0.00013
***
P = 0.07519
n.s.
P = 0.08769
n.s.
BRG1
P = 0.00004
****
P = 0.00067
***
P = 0.04927
*
(upper bands)
P = 0.00202
**
P = 0.08122
n.s.
P = 0.10149
n.s.
(lower bands)
BRD7
P = 0.00439
**
P = 0.00670
**
P = 0.12192
n.s.
PHF10
P = 0.02739
*
P = 0.06212
n.s.
P = 0.04763
*
1.0
0.5
0.0
CT8
CT20
PerKO

Figure 3a

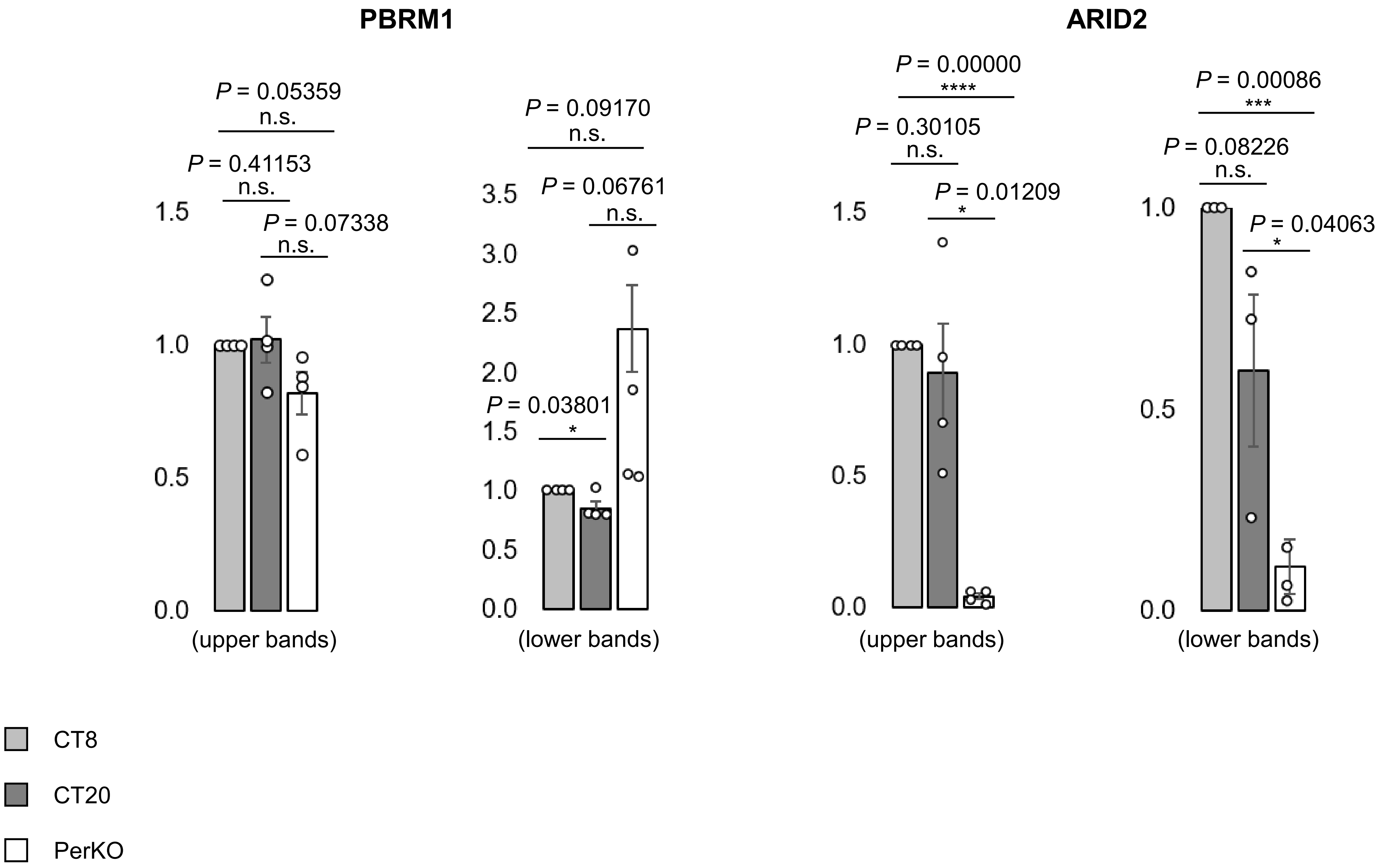

Figure 3b, S5a

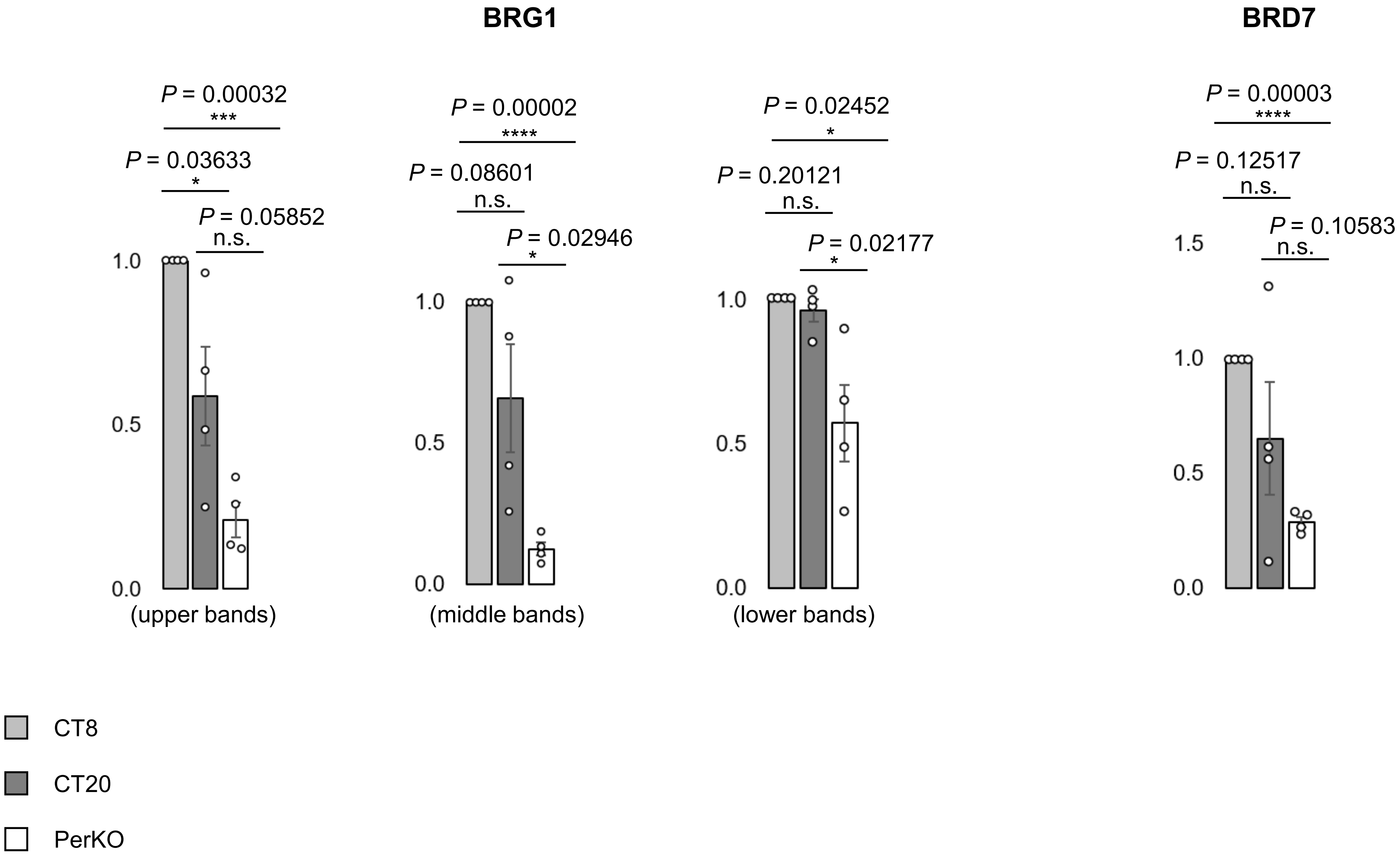

Figure 3b, S5a

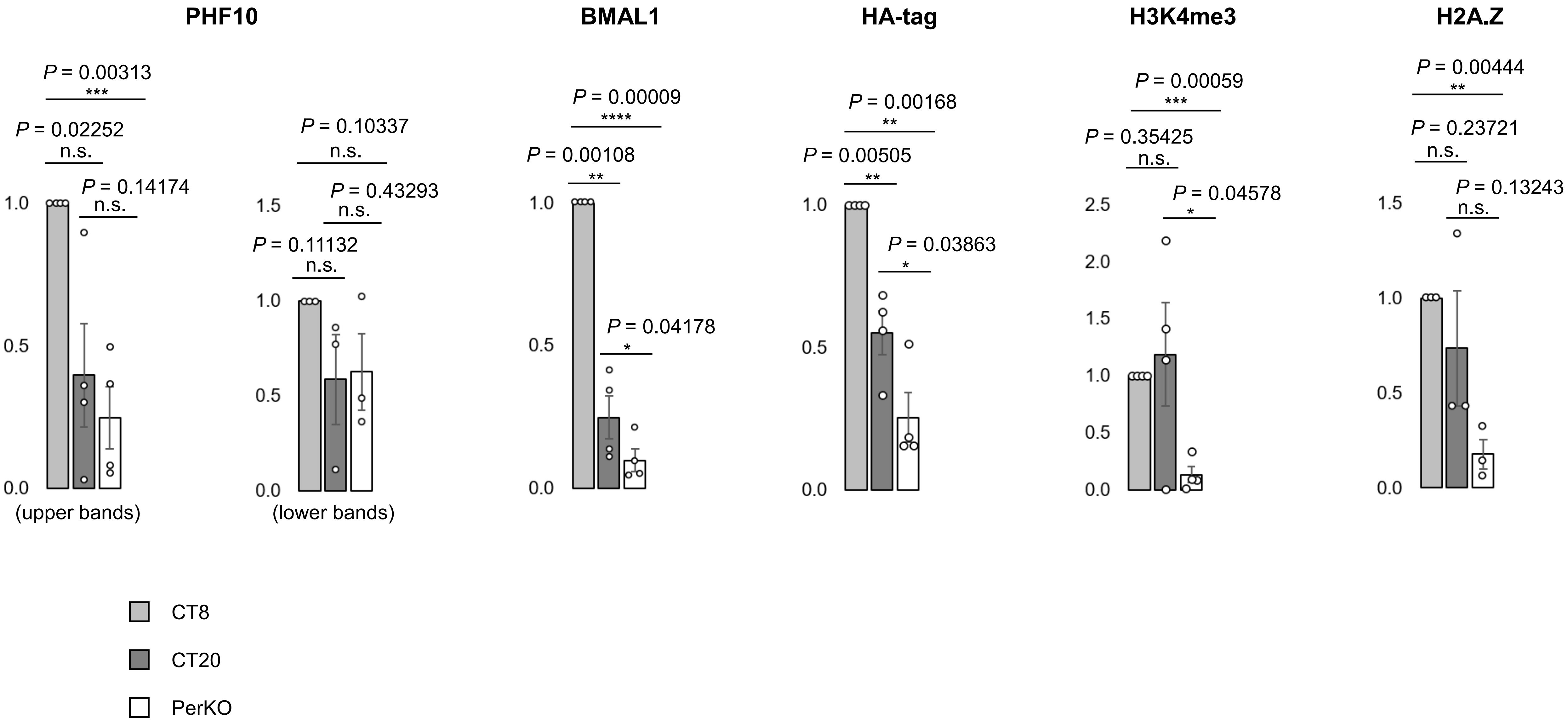

Figure 3b, S5a

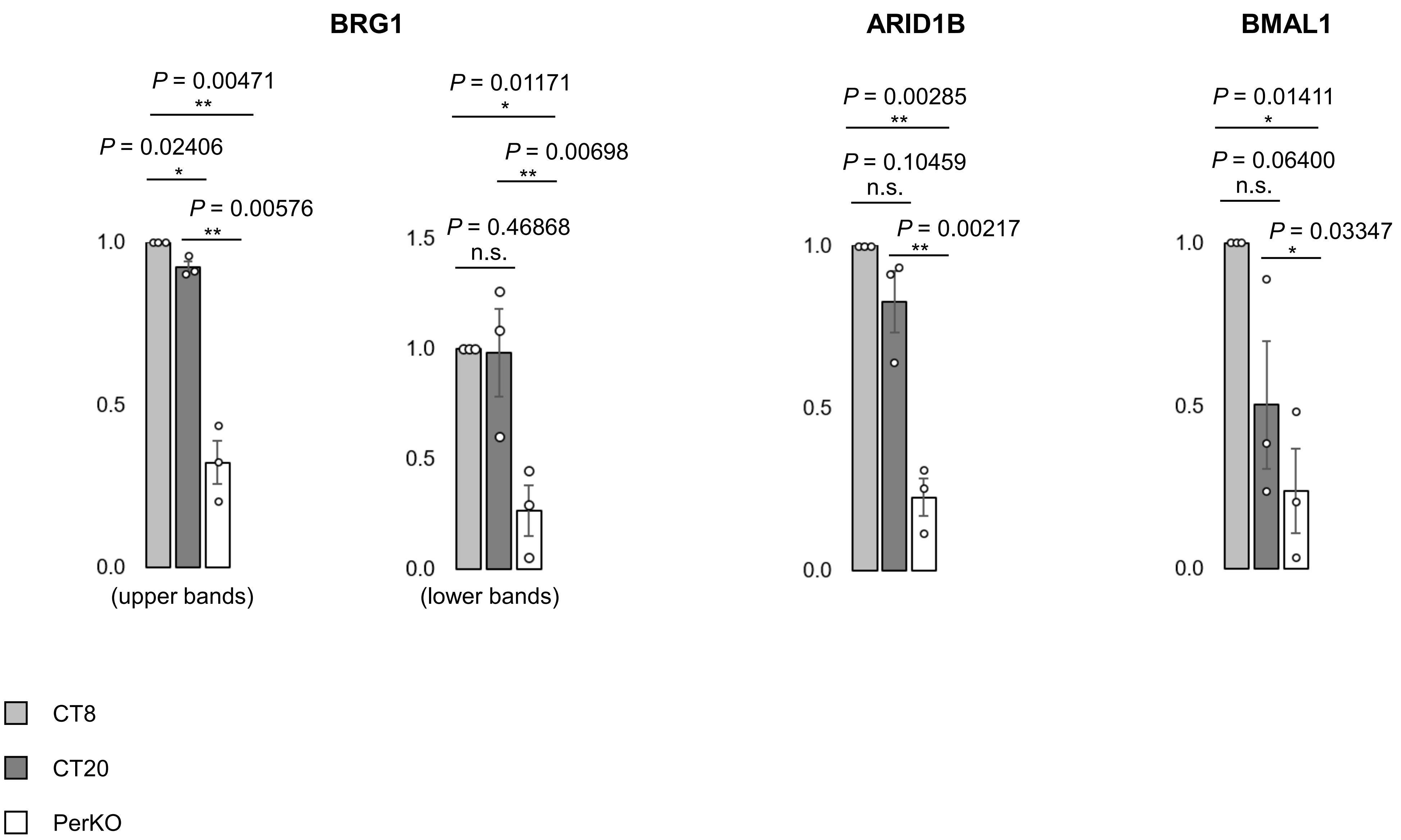
BRG1
P = 0.00471
**
P = 0.02406
*
P = 0.00576
**
1.0
0.5
0.0
(upper bands)
P = 0.01171
*
P = 0.00698
**
P = 0.46868
n.s.
1.5
1.0
0.5
0.0
(lower bands)
ARID1B
P = 0.00285
**
P = 0.10459
n.s.
P = 0.00217
**
1.0
0.5
0.0
BMAL1
P = 0.01411
*
P = 0.06400
n.s.
P = 0.03347
*
1.0
0.5
0.0
CT8
CT20
PerKO

Figure 4a

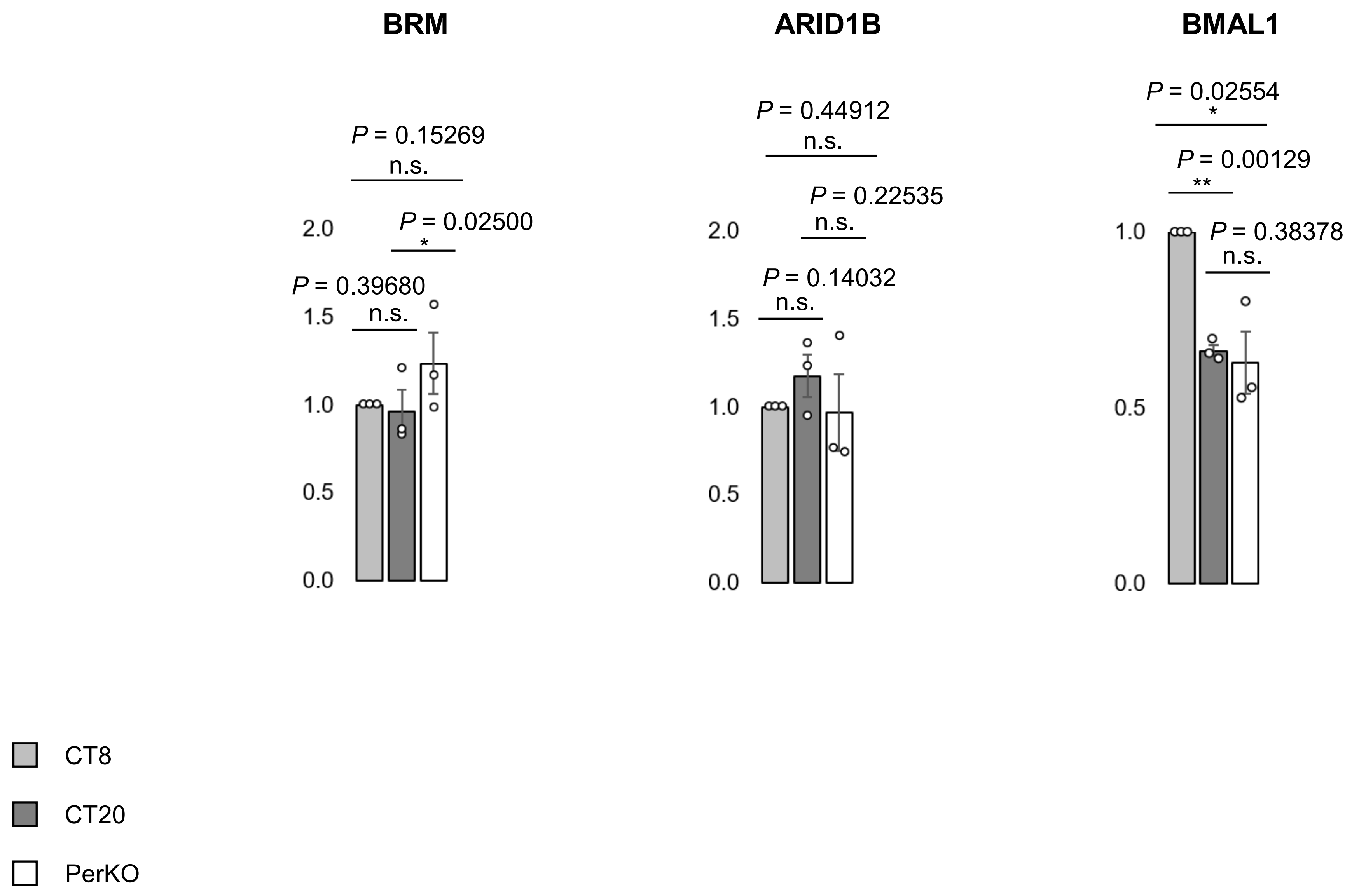
BRM
ARID1B
BMAL1
P = 0.15269
n.s.
P = 0.02500
*
P = 0.39680
n.s.
P = 0.44912
n.s.
P = 0.22535
n.s.
P = 0.14032
n.s.
P = 0.02554
*
P = 0.00129
**
P = 0.38378
n.s.
0.0
0.5
1.0
1.5
2.0
CT8
CT20
PerKO

Figure 4b

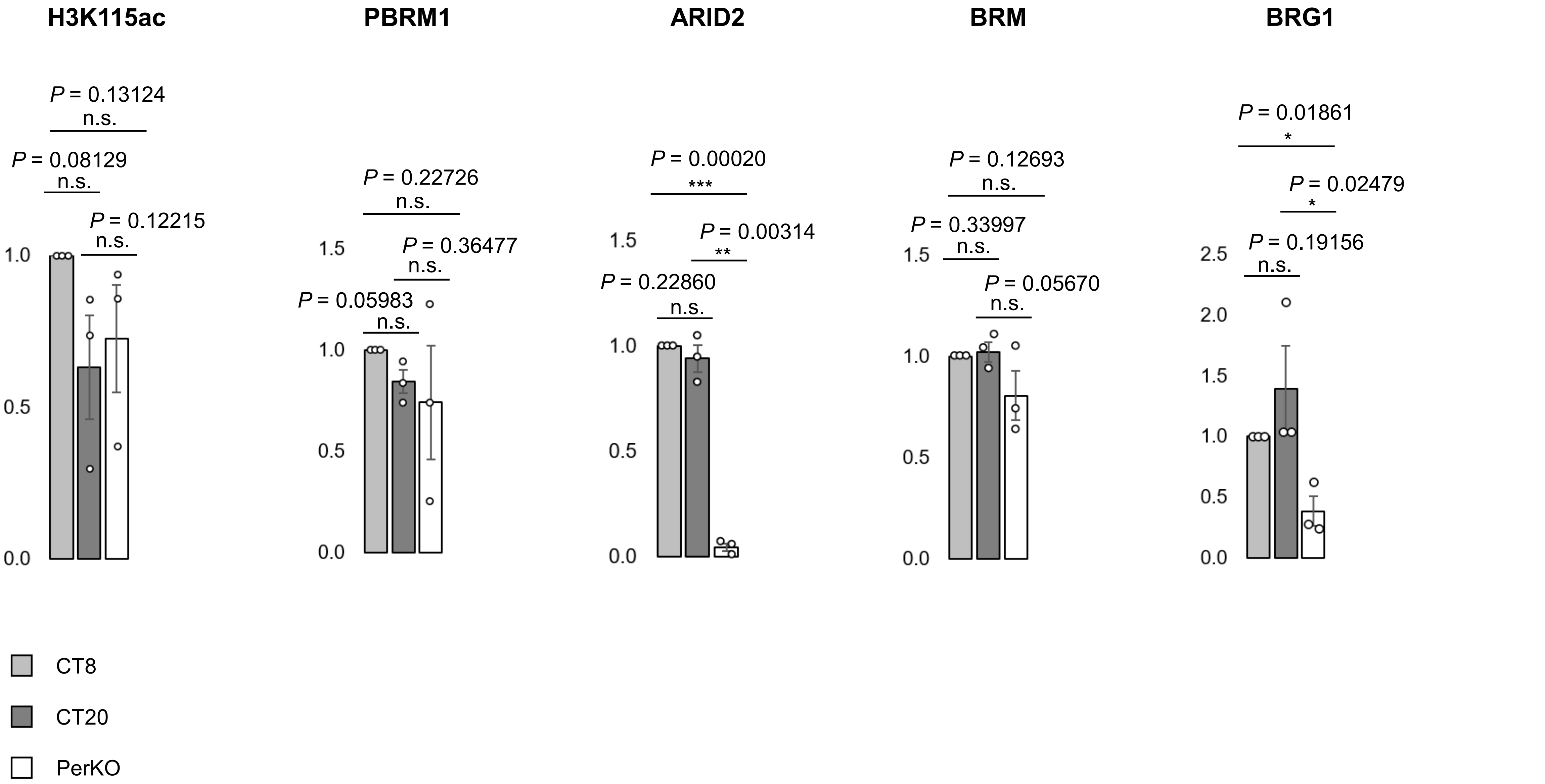
H3K115ac
P = 0.13124
n.s.
P = 0.08129
n.s.
P = 0.12215
n.s.
1.0
0.5
0.0
PBRM1
P = 0.22726
n.s.
P = 0.36477
n.s.
P = 0.05983
n.s.
1.5
1.0
0.5
0.0
ARID2
P = 0.00020
***
P = 0.00314
**
P = 0.22860
n.s.
1.5
1.0
0.5
0.0
BRM
P = 0.12693
n.s.
P = 0.33997
n.s.
P = 0.05670
n.s.
1.5
1.0
0.5
0.0
BRG1
P = 0.01861
*
P = 0.02479
*
P = 0.19156
n.s.
2.5
2.0
1.5
1.0
0.5
0.0
CT8
CT20
PerKO

Figure 5b

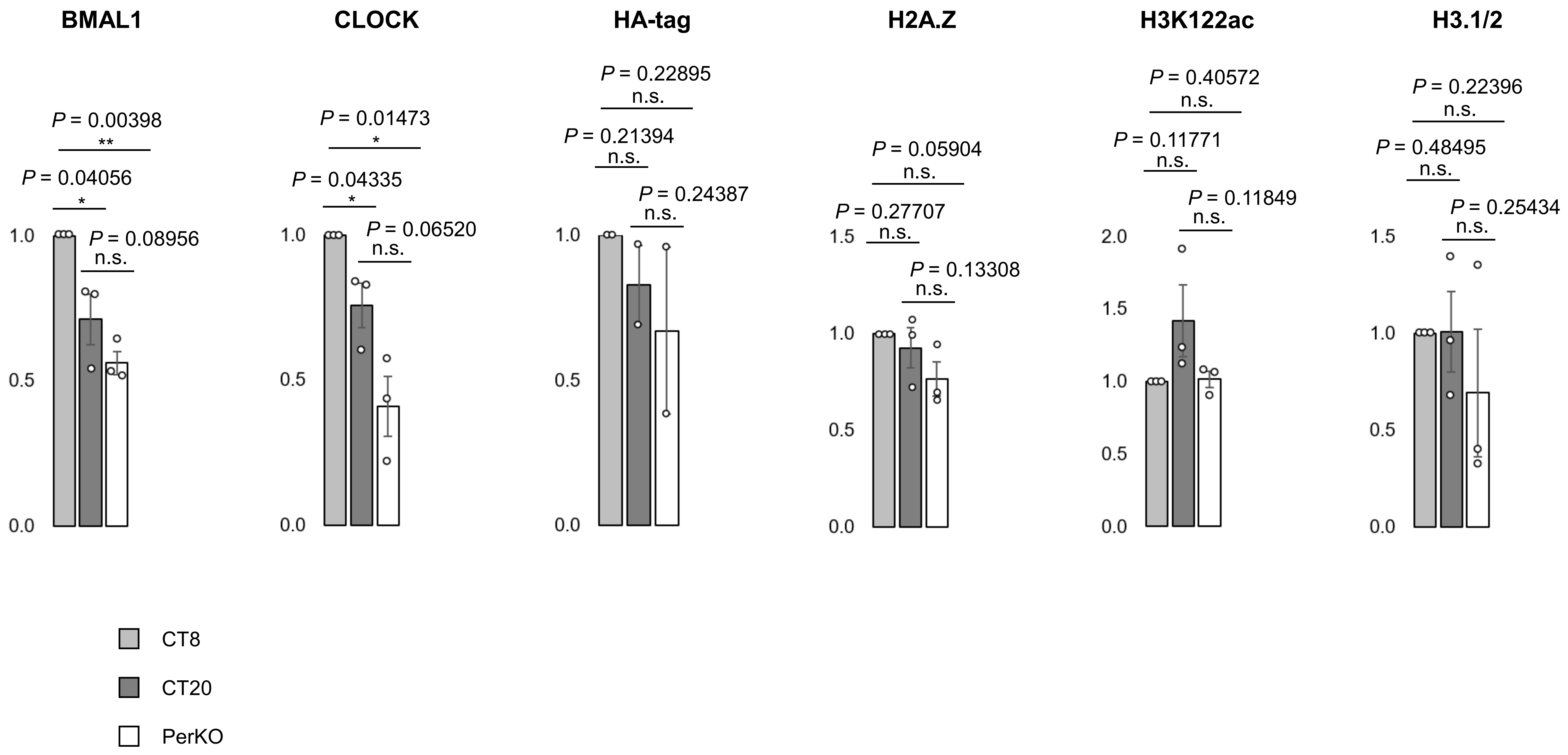
BMAL1
CLOCK
HA-tag
H2A.Z
H3K122ac
H3.1/2
P = 0.00398
**
P = 0.04056
*
P = 0.08956
n.s.
P = 0.01473
*
P = 0.04335
*
P = 0.06520
n.s.
P = 0.22895
n.s.
P = 0.21394
n.s.
P = 0.24387
n.s.
P = 0.05904
n.s.
P = 0.27707
n.s.
P = 0.13308
n.s.
P = 0.40572
n.s.
P = 0.11771
n.s.
P = 0.11849
n.s.
P = 0.22396
n.s.
P = 0.48495
n.s.
P = 0.25434
n.s.
CT8
CT20
PerKO

Figure 5b

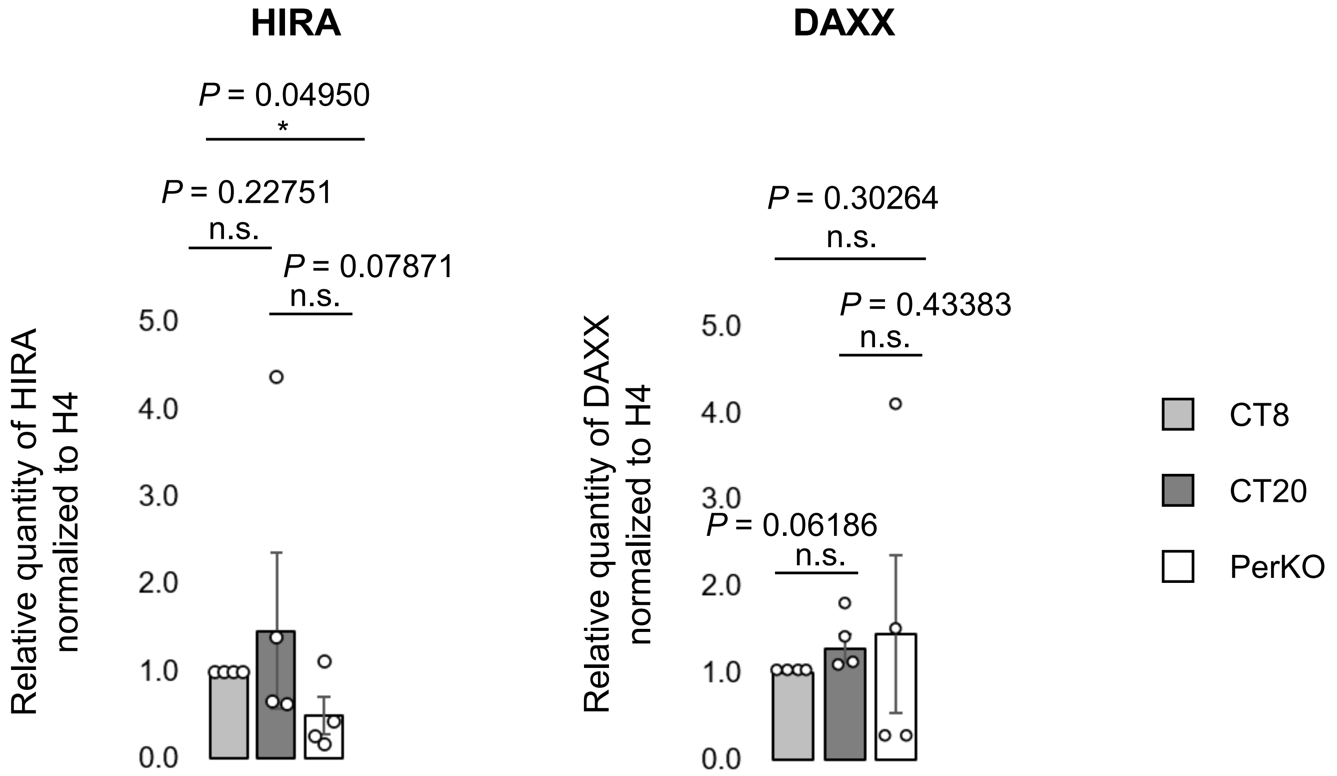
HIRA
DAXX
P = 0.04950
*
P = 0.22751
n.s.
P = 0.07871
n.s.
P = 0.30264
n.s.
P = 0.43383
n.s.
P = 0.06186
n.s.
Relative quantity of HIRA normalized to H4
Relative quantity of DAXX normalized to H4
5.0
4.0
3.0
2.0
1.0
0.0
CT8
CT20
PerKO

Figure S6b
